# Supplementary material for: TidyMass an object-oriented reproducible analysis framework for LC–MS data
Source: Nat Commun. 2022 Jul 28;13:4365. doi: 10.1038/s41467-022-32155-w (PMC9334349; doi:10.1038/s41467-022-32155-w)
Supplement: Supplementary file 1 — Supplementary information [file 41467_2022_32155_MOESM1_ESM.pdf]

Supplementary Information: TidyMass An Object-oriented Reproducible Analysis Framework for LC-MS Data

M. Snyder et al.

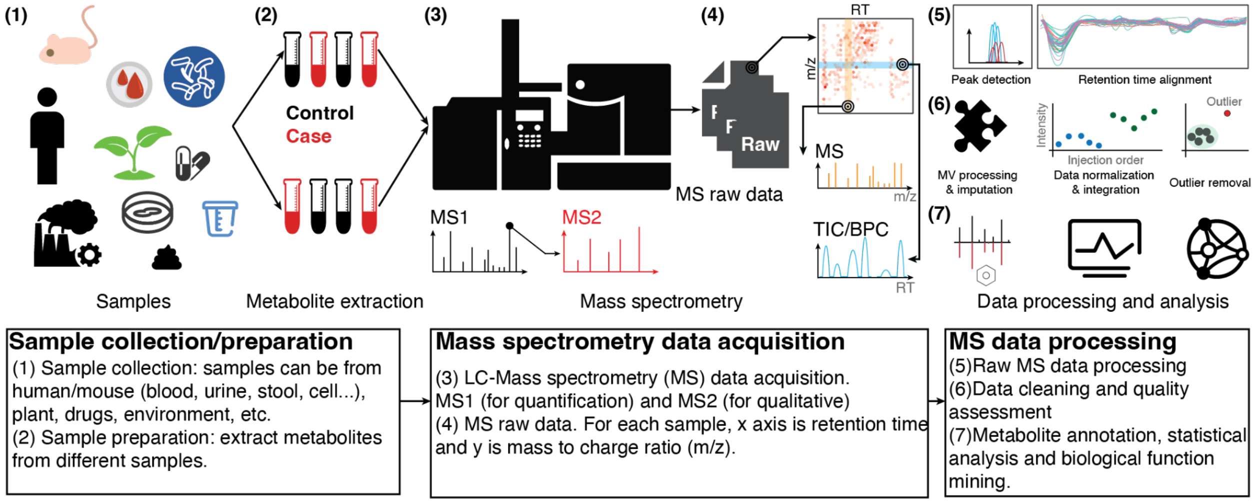

Supplementary Figure 1. Example of an untargeted high-resolution LC-MS experiment. A diagram of an experimental and analytical workflow for LC-MS-based untargeted metabolomics. LC-MS-based untargeted metabolomics involves several fundamental steps: **(1)** sample collection and preparation; **(2)** metabolite extraction; **(3), (4)** mass spectrometry data acquisition and raw data generation; **(5)** raw MS data processing; **(6)** data cleaning and quality assessment; and **(7)** metabolite annotation, statistical analysis and biological function mining. The intended role of *tidymass* is in MS data processing and analysis (steps 5-7). Icons used in this figure are from www.iconfont.cn/.

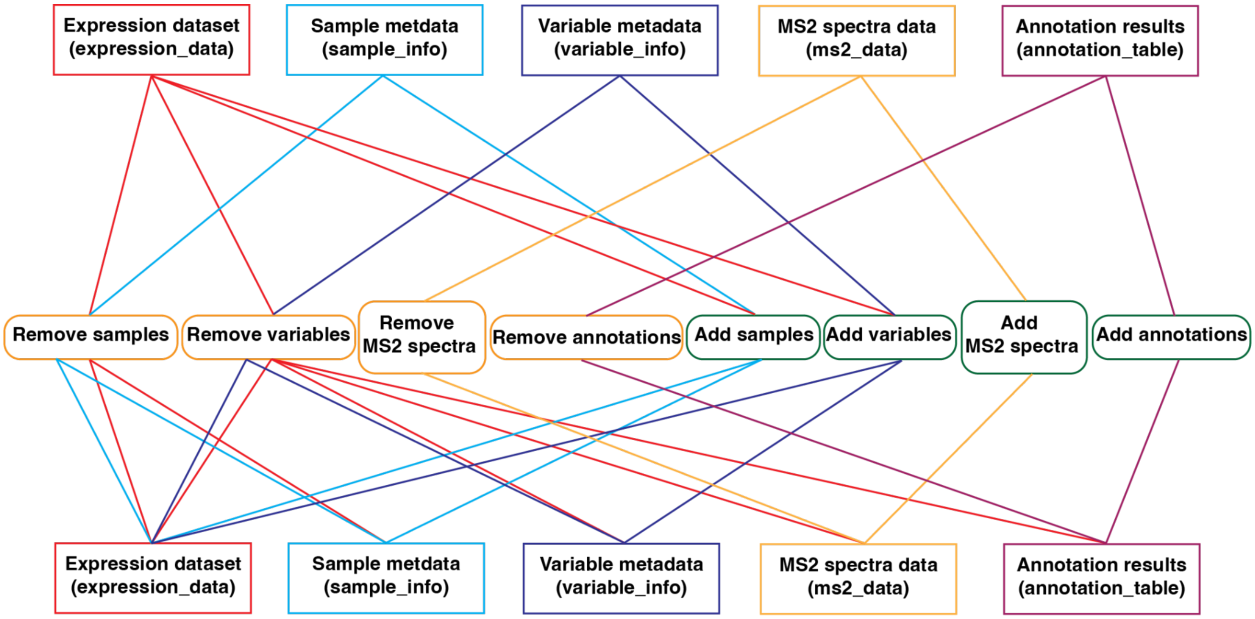

Supplementary Figure 2. Functions that support the "mass_dataset" class. (a) Functions to extract and export data. **(b)** Functions to summarize and explore data. **(c)** Functions for preprocessing data. (d) Functions to combine/merge data.

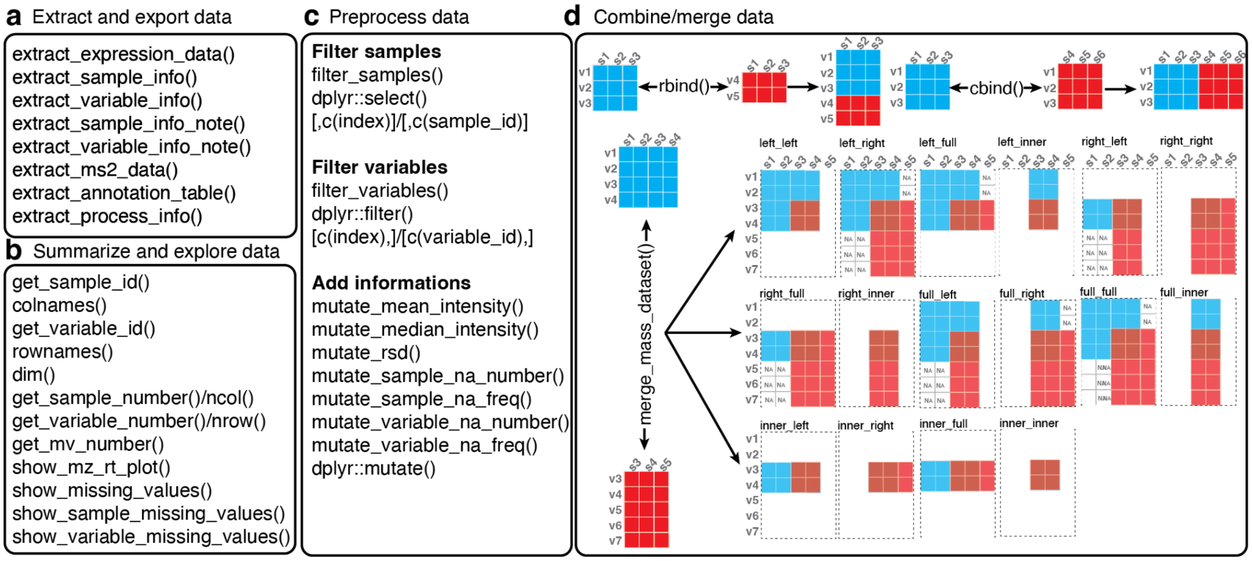

Supplementary Figure 3. Functions that support the "mass_dataset" class. (a) Functions to extract and export data. **(b)** Functions to summarize and explore data. **(c)** Functions for preprocessing data. (d) Functions to combine/merge data.

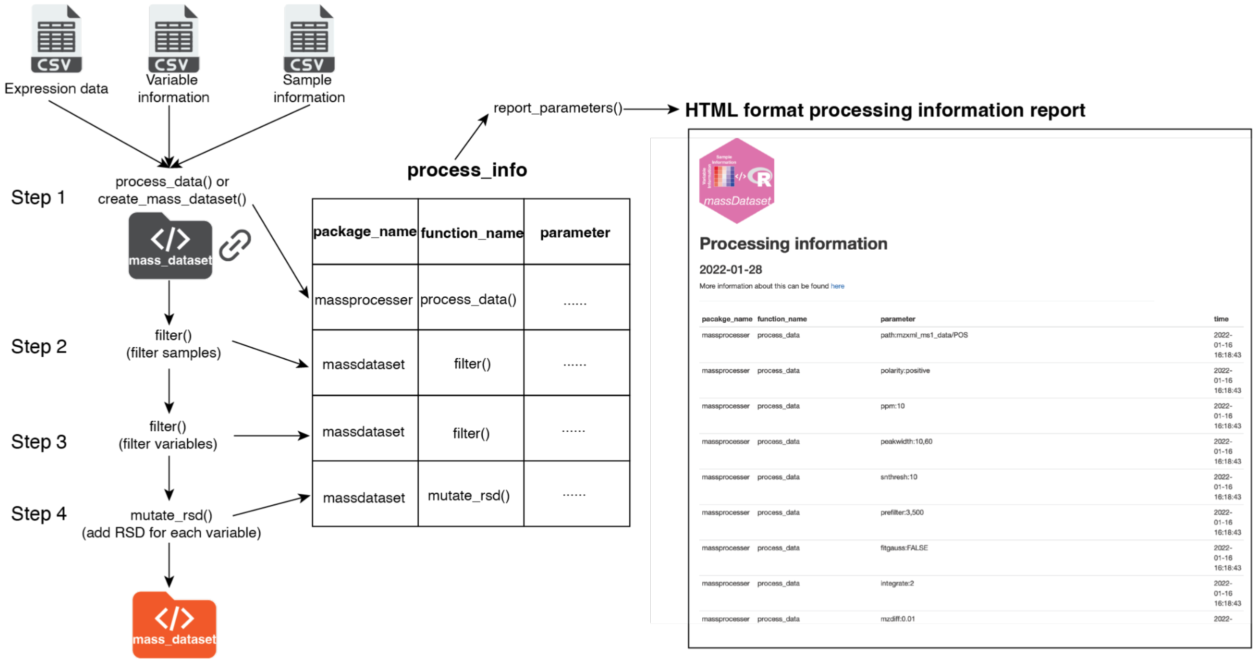

Supplementary Figure 4. Processing information in the "mass_dataset" class. Different functions from different packages can apply to the "mass_dataset" class step by step, and the parameters will be recorded in the "process_info" slot. The "report_parameters()" function from the massDataset package can be used to extract them and output them as an HTML format processing information report. Icons used in this figure are from www.iconfont.cn/.

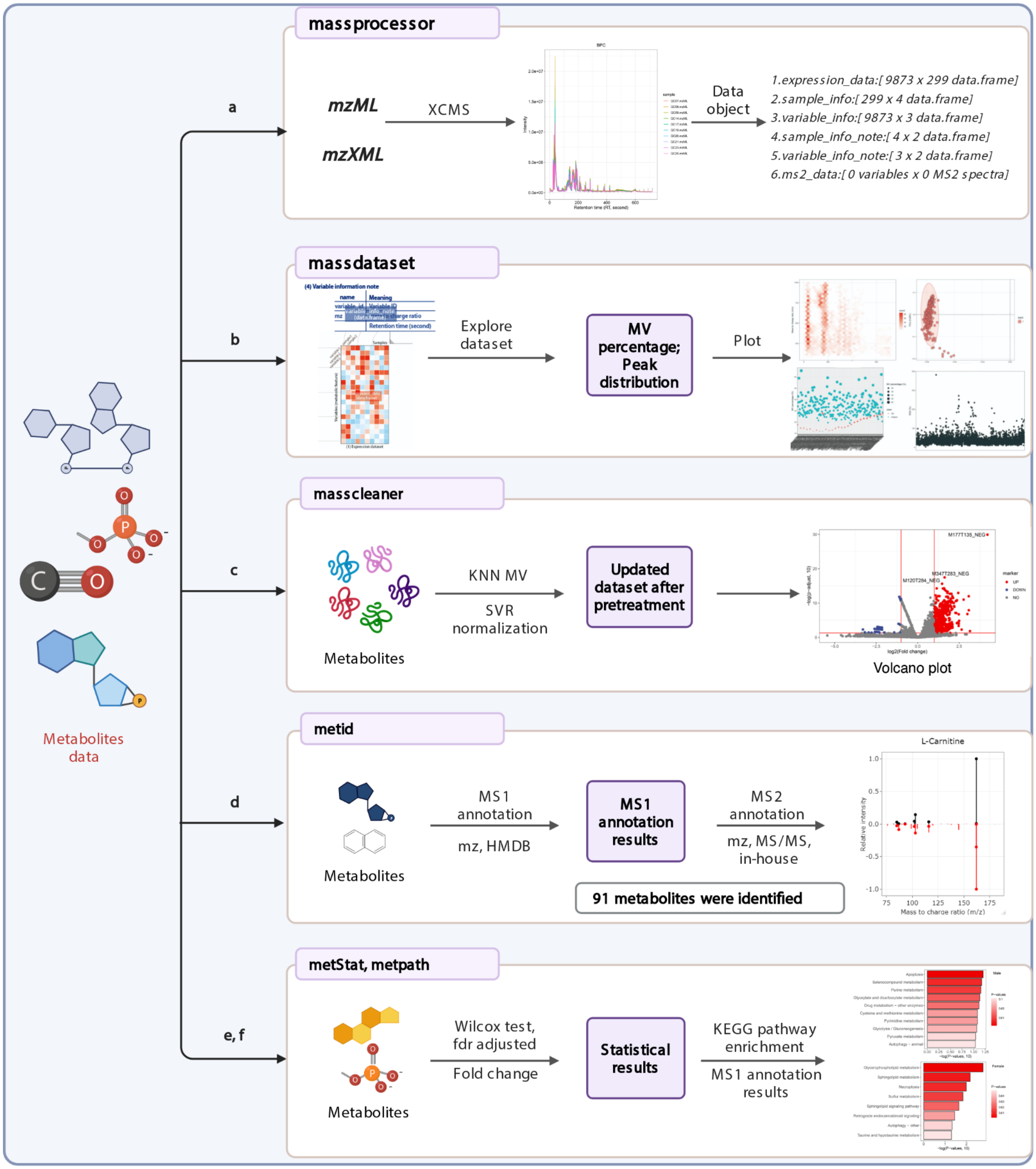

Supplementary Figure 5. Analysis workflow, an example case study. **(a)**, **(b)**, **(c)**, **(d)**, **(e)**, and **(f)** correspond to the step in **Fig. 2**. This figure is created with BioRender.com.

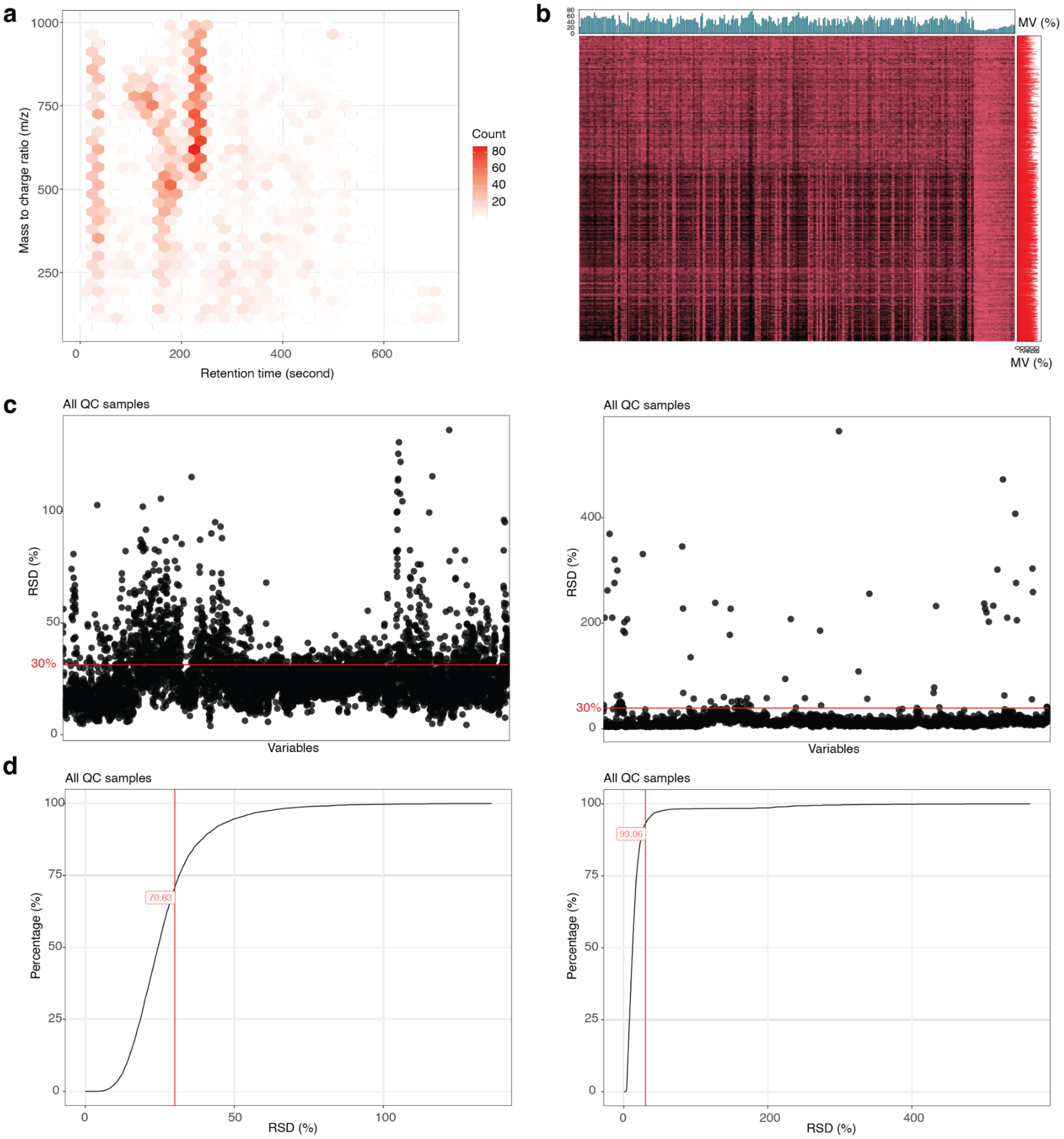

Supplementary Figure 6. Data quality assessment before and after data cleaning using tidyMass. Here the HILIC positive mode data is used as an example. **(a)** The distribution of metabolic features. **(b)** Missing value distribution. The X-axis displays samples, and the y-axis displays metabolic features. **(c)** RSD for all the metabolic features in QC samples before (left) and after data cleaning (right). **(d)** RSD cumulative plot before (left) and after data cleaning (right).

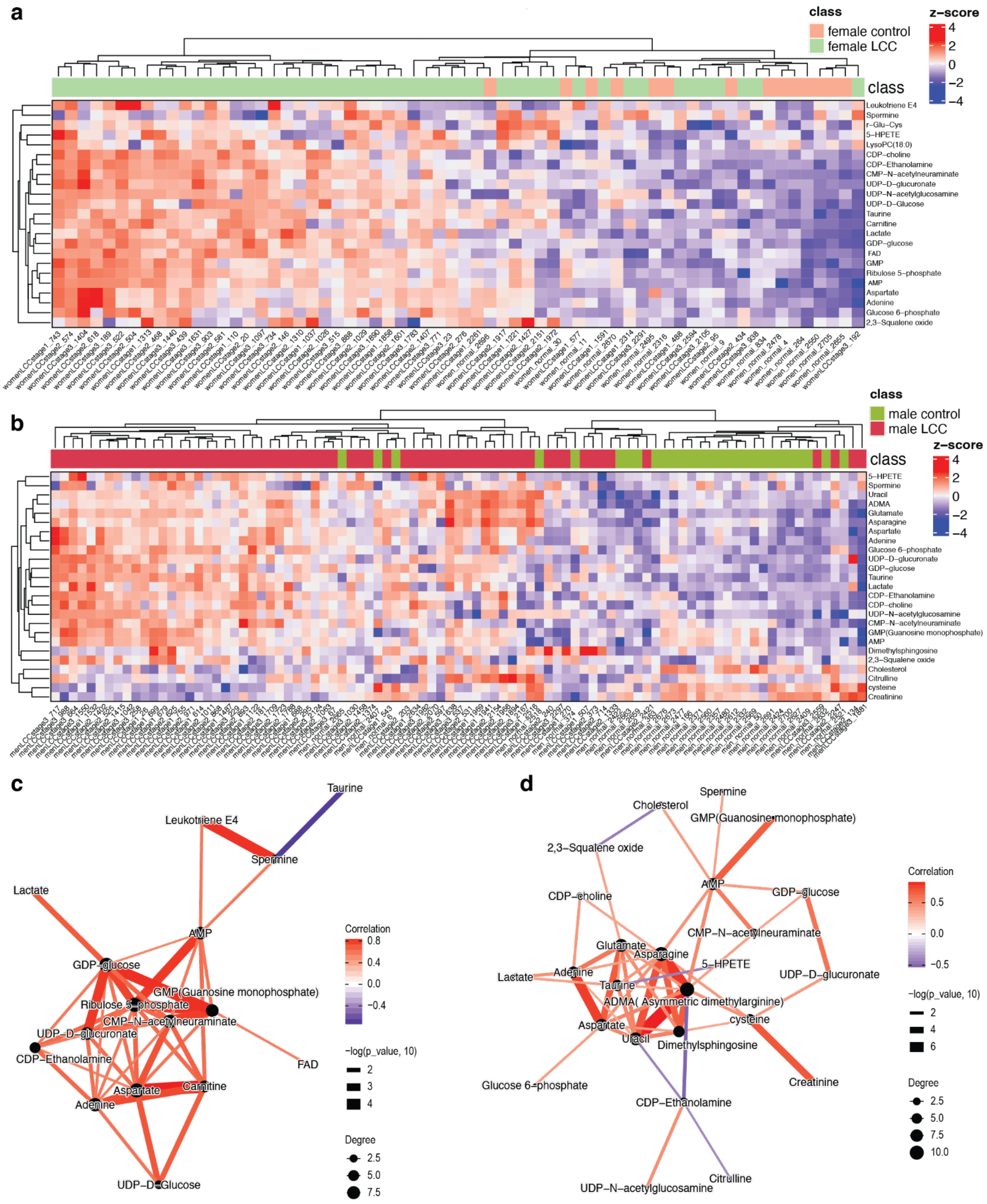
a
class
female control
female LCC
z-score
4
2
0
−2
−4
class
Leukotriene E4
Spermine
r−Glu−Cys
5−HPETE
LysoPC(18:0)
CDP−choline
CDP−Ethanolamine
CMP−N−acetylneuraminate
UDP−D−glucuronate
UDP−N−acetylglucosamine
UDP−D−Glucose
Taurine
Carnitine
Lactate
GDP−glucose
FAD
GMP
Ribulose 5−phosphate
AMP
Aspartate
Adenine
Glucose 6−phosphate
2,3−Squalene oxide
b
class
male control
male LCC
z-score
4
2
0
−2
−4
class
5−HPETE
Spermine
Uracil
ADMA
Glutamate
Asparagine
Aspartate
Adenine
Glucose 6−phosphate
UDP−D−glucuronate
GDP−glucose
Taurine
Lactate
CDP−Ethanolamine
CDP−choline
UDP−N−acetylglucosamine
CMP−N−acetylneuraminate
GMP(Guanosine monophosphate)
AMP
Dimethylsphingosine
2,3−Squalene oxide
Cholesterol
Citrulline
cysteine
Creatinine
c
Taurine
Leukotriene E4
Spermine
Lactate
AMP
GDP−glucose
GMP(Guanosine monophosphate)
Ribulose 5−phosphate
CMP−N−acetylneuraminate
UDP−D−glucuronate
CDP−Ethanolamine
FAD
Aspartate
Carnitine
Adenine
UDP−D−Glucose
Correlation
0.8
0.4
0.0
−0.4
−log(p_value, 10)
2
3
4
Degree
2.5
5.0
7.5
d
Spermine
Cholesterol
GMP(Guanosine monophosphate)
2,3−Squalene oxide
AMP
CDP−choline
GDP−glucose
CMP−N−acetylneuraminate
Glutamate
Asparagine
5−HPETE
Lactate
Adenine
UDP−D−glucuronate
Taurine
ADMA(Asymmetric dimethylarginine)
Aspartate
cysteine
Uracil
Dimethylsphingosine
Creatinine
Glucose 6−phosphate
CDP−Ethanolamine
Citrulline
UDP−N−acetylglucosamine
Correlation
0.5
0.0
−0.5
−log(p_value, 10)
2
4
6
Degree
2.5
5.0
7.5
10.0

Supplementary Figure 7. Differentially expressed metabolites (DEMs) for tumors from female and male patients with left-sided colorectal cancer (LCC). (a) Heatmap for normal colon (control) and tumor tissues from female patients using DEMs. **(b)** Heatmap for normal colon (control) and tumor tissues from male patients using DEMs. **(c)** Spearman correlation networks for metabolites dysregulated in tumors compared to normal tissues from female **(c)** and male patients **(d)**. The degree means the connection numbers of one node.

Supplementary Note 1

The quantitative assessments of different aspects were made according to the previous publications[1,2] for these tools and our experience (**Supplementary Table 1**). The detailed explanations for the quantitative assessments are provided as follows:

"*Cross-platform utility*" evaluated whether the tool can be used on different platforms, such as Windows, Linux, and Mac OS. If the tool can be used on all three platforms, this aspect is labeled as "+++". If the tool can only be used on one platform, this aspect is represented as "+". For example, tidyMass can be used in Windows, Linux, and Mac OS, so it is labeled as "+++" in this criterion.

"*Uniform object*" evaluates whether the tool has a uniform data format for the whole pipeline. This criterion is only for command-line tools. For the GUI/online tools, no uniform data format is used, so they are labeled as "-" in this criterion. For example, for the "RforMassSpectrometry", multiple infrastructures are used in the whole pipeline ("MsExperiment", "Spectra", and "QFeatures"), so it is labeled as "+" in this criterion. For metaboanalystR, the "mSet" class is used in the whole workflow, however, this class can't include processing history and arguments, so it is labeled as "+".

"*Object-oriented pipeline*" evaluates whether the tool provides a pipeline that is based on the concept of "objects", which can contain data and code. So this criterion is only for command-line tools, and the tools should have and uniform object for input. For example, there is no uniform object for patRoon, so it is labeled as "-".

"*Module design*" evaluates whether the tool adopts the module design concept. Module design means that if the functions in the tools are grouped into different "modules" (for R/python, they are packages). For example, RforMassSpectrometry, all the functions for metabolite annotation are in the "MetaboAnnotation" package, and all the functions for the compound databases are in the "CompoundDb" package. So it is labeled as "✓".

"*Integrated with tidyverse*" evaluates whether the tool supports the functions in the "tidyverse" package, which is one of the most popular tools for data science in the R environment. So this criterion is only for R tools. For tidyMass, the "mass_dataset" class supports most of the functions in the "tidyverse" package. For example, the "filter" function in "tidyverse" could be used to filter samples/variables in "mass_dataset", so this criterion is labeled as "✓" for tidyMass.

"*Support existing tools*" evaluates whether the tool supports the functions of other tools. This criterion is only for R tools. For example, in tidyMass, the "mass_dataset" class supports the functions from "complexHeatmap"[3], "mixOmics"[4], and R base functions.

The last six criteria, including "*Peak picking & alignment*", "*Data cleaning*", "*Metabolite annotation*", "*Statistical analysis*", "*Pathway enrichment*" and "*Quality assessment*", evaluate whether the tool has these functions. If the tool has the function, the aspect is labeled as "√", and if the tool does not has the function, the aspect is labeled as "-".

Supplementary Table 1. Comparison between tidyMass and other tools.

Name	tidyMass	RforMassSpectrometry[5]	metaboanalystR[6]	openMS[7]	Galaxy-M[8]	NP analyst[9]	patRoon[10]	eMZed[11]
Software type	1	1	1	1	1	2	1	1
Cross-platform utility	+++	+++	+++	+++	+++	+++	+++	+++
Uniform object	+++	+	+	-	-	-	-	-
Object-oriented pipeline	+++	+	+	-	-	-	-	-
Module design	✓	✓	-	✓	-	-	-	-
Integrated with tidyverse	✓	-	-	-	-	-	-	-
Support existing tools	✓	-	-	-	-	-	✓	-
Peak picking & alignment	✓	✓	✓	✓	✓	-	✓	✓
Data cleaning	✓	-	✓	-	✓	-	✓	-
Metabolite annotation	✓	✓	✓	✓	✓	✓	✓	+
Statistical analysis	✓	-	✓	-	✓	-	-	-
Pathway enrichment	✓	-	✓	-	-	-	-	-
Quality assessment	✓	-	✓	-	-	-	✓	-

Note: Symbols used for feature evaluations with "√" for present, "-" for absent, and "+" for a more quantitative assessment, with more "+" indicating better support. For software type, 1 represents the command-line tool, and 2 represents GUI/online tool.

Supplementary Table 2. Parameters for conversion using massConvereter.

Argument name	Meaning	Format	Range
output_format	Output format	character	mzXML, mzXL, mz5, mgf, text, ms1, cms1, ms2, cms2
binary_encoding_precision	Binary encoding precision	character	32 or 64
zlib = TRUE	Zlib or not	logical	TURE or FALSE
write_index	Write index or not	logical	TRUE or FALSE
peak_picking_algorithm	Peak picking algorithm	character	vendor, cwt, no
vendor_mslevels	Vender MS levels	vector	1-n
cwt_mslevels	Cwt MS level	vector	1-n
cwt_min_snr	Cwt minimum signal to noise rate	numeric	0-1
cwt_min_peak_spacing	Cwt minimum peak spacing	numeric	0-1
subset_polarity	Subset polarity	character	any, positive, negative
subset_scan_number	Subset scan number	vector	0-n
subset_scan_time	Subset scan time	vector	0-n
subset_mslevels	Subset MS level	vector	1–n
zero_samples_mode	Zero sample mode	character	no, removeExtra, addMissing
zero_samples_mslevels	Zero sample MS levels	vector	1-n
zero_samples_add_missing_flanking_zero_count	Zero sample add missing flanking zero count	numeric	0-10

Supplementary References

1. Wen, B., Mei, Z., Zeng, C. & Liu, S. metaX: a flexible and comprehensive software for processing metabolomics data. *BMC Bioinformatics* **18**, 183 (2017).
2. Chong, J. *et al.* MetaboAnalyst 4.0: towards more transparent and integrative metabolomics analysis. *Nucleic Acids Research* vol. 46 W486–W494 (2018).
3. Gu, Z., Eils, R. & Schlesner, M. Complex heatmaps reveal patterns and correlations in multidimensional genomic data. *Bioinformatics* **32**, 2847–2849 (2016).
4. Rohart, F., Gautier, B., Singh, A. & Cao, K.-A. L. mixOmics: An R package for 'omics feature selection and multiple data integration. *PLOS Computational Biology* vol. 13 e1005752 (2017).
5. Rainer, J. *et al.* A Modular and Expandable Ecosystem for Metabolomics Data Annotation in R. *Metabolites* vol. 12 173 (2022).
6. Pang, Z., Chong, J., Li, S. & Xia, J. MetaboAnalystR 3.0: Toward an Optimized Workflow for Global Metabolomics. *Metabolites* vol. 10 186 (2020).
7. Sturm, M. *et al.* OpenMS - an open-source software framework for mass spectrometry. *BMC Bioinformatics* **9**, 163 (2008).
8. Davidson, R. L., Weber, R. J. M., Liu, H., Sharma-Oates, A. & Viant, M. R. Galaxy-M: a Galaxy workflow for processing and analyzing direct infusion and liquid chromatography mass spectrometry-based metabolomics data. *Gigascience* **5**, 10 (2016).
9. Lee, S. *et al.* NP Analyst: An Open Online Platform for Compound Activity Mapping. *ACS Cent Sci* **8**, 223–234 (2022).
10. Helmus, R., Ter Laak, T. L., van Wezel, A. P., de Voogt, P. & Schymanski, E. L. patRoon: open source software platform for environmental mass spectrometry based non-target screening. *J. Cheminform.* **13**, 1 (2021).
11. Kiefer, P., Schmitt, U. & Vorholt, J. A. eMZed: an open source framework in Python for rapid and interactive development of LC/MS data analysis workflows. *Bioinformatics* **29**, 963–964 (2013).

12. Cai, Y. *et al.* Sex Differences in Colon Cancer Metabolism Reveal A Novel Subphenotype. *Sci. Rep.* **10**, 4905 (2020).
