# Supplementary material for: TidyMass an object-oriented reproducible analysis framework for LC–MS data
Source: Nat Commun. 2022 Jul 28;13:4365. doi: 10.1038/s41467-022-32155-w (PMC9334349; doi:10.1038/s41467-022-32155-w)
Supplement: Supplementary file 2 — Description of Additional Supplementary Files [file 41467_2022_32155_MOESM2_ESM.docx]

File name: **Supplementary Data 1**

Description: Functions in the tidyMass project for data processing and analysis.

File name: **Supplementary Data 2**

Description: Processed data (“mass_dataset” class) from the massProcesser package.

File name: **Supplementary Data 3**

Description: Code file (Rmd format) for the case study.
