# Supplementary material for: TidyMass an object-oriented reproducible analysis framework for LC–MS data
Source: Nat Commun. 2022 Jul 28;13:4365. doi: 10.1038/s41467-022-32155-w (PMC9334349; doi:10.1038/s41467-022-32155-w)
Supplement: Supplementary file 3 — Dataset 1 [file 41467_2022_32155_MOESM3_ESM.docx]

**Supplementary Data 1.** Functions in the tidyMass project for data processing and analysis.

| **Function name** | **package** | **Input data format** | **Output** | **Function** |
| --- | --- | --- | --- | --- |
| docker_pull_pwiz() | massConverter | - | - | Pull pwiz docker image. |
| create_msconvert_parameter() | massConverter | - | msconvert_parameter | Create parameter class for massConverter. |
| convert_raw_data() | massConverter | Mass spectrometry raw data | Other format data | Convert MS raw data to other format data |
| create_mass_dataset() | massDataset | - | “mass_dataset” class object | Create “mass_dataset” class object |
| extract_expression_data() | massDataset | “mass_dataset” class object | data.frame | Extract expression data |
| extract_sample_info() | massDataset | “mass_dataset” class object | data.frame | Extract sample information |
| extract_variable_info() | massDataset | “mass_dataset” class object | data.frame | Extract variable information |
| extract_annotation_table() | massDataset | “mass_dataset” class object | data.frame | Extract annotation table |
| extract_variable_info_note() | massDataset | “mass_dataset” class object | data.frame | Extract variable information metadata |
| extract_sample_info_note() | massDataset | “mass_dataset” class object | data.frame | Extract sample information metadata |
| extract_process_info() | massDataset | “mass_dataset” class object | “tidymass_parameter” class | Extract processing information |
| extract_ms2_data() | massDataset | “mass_dataset” class object | “ms2_data” class | Extract MS^2^ data |
| mz_rt_match() | massTools | “mass_dataset” class object | data.frame | Match two “mass_dataset” class according to mz and rt |
| filter_samples() | massDataset | “mass_dataset” class object | “mass_dataset” class object | Filter samples |
| filter_variables() | massDataset | “mass_dataset” class object | “mass_dataset” class object | Filter variables |
| filter() | dplyr | “mass_dataset” class object | “mass_dataset” class object | Filter rows from components in “mass_dataset” class |
| mutate() | dplyr | “mass_dataset” class object | “mass_dataset” class object | Mutate new columns to components in “mass_dataset” class |
| select() | dplyr | “mass_dataset” class object | “mass_dataset” class object | Select columns from components in “mass_dataset” class |
| mutate_ms2() | massDataset | “mass_dataset” class object | “mass_dataset” class object | Add MS^2^ data to “mass_dataset” class |
| mutate_mean_intensity() | massDataset | “mass_dataset” class object | “mass_dataset” class object | Add mean intensity to variable information |
| mutate_median_intensity() | massDataset | “mass_dataset” class object | “mass_dataset” class object | Add median intensity to variable information |
| mutate_rsd() | massDataset | “mass_dataset” class object | “mass_dataset” class object | Add RSD to variable information |
| mutate_sample_na_number() | massDataset | “mass_dataset” class object | “mass_dataset” class object | Add variable NA numbers to sample information |
| mutate_sample_na_freq() | massDataset | “mass_dataset” class object | “mass_dataset” class object | Add variable NA frequency to sample information |
| mutate_sample_na_number() | massDataset | “mass_dataset” class object | “mass_dataset” class object | Add sample NA numbers to variable information |
| mutate_sample_na_freq() | massDataset | “mass_dataset” class object | “mass_dataset” class object | Add sample NA frequency to variable information |
| report_parameters() | massDataset | “mass_dataset” class object | HTML format report | Report for processing information |
| show_mz_rt_plot() | massDataset | “mass_dataset” class object | ggplot2 plot class object | Metabolic feature plot |
| show_missing_values() | massDataset | “mass_dataset” class object | ggplot2 plot class object | Missing value distribution plot |
| left_join() | dplyr | “mass_dataset” class object | “mass_dataset” class object | Mutate new columns to components in “mass_dataset” class |
| cbind() | base | “mass_dataset” class object | “mass_dataset” class object | Bind two “mass_dataset” class objects by rows |
| rbind() | base | “mass_dataset” class object | “mass_dataset” class object | Bind two “mass_dataset” class objects by columns |
| merge_mass_dataset() | massDataset | “mass_dataset” class object | “mass_dataset” class object | Merge two “mass_dataset” class objects |
| process_data() | massProcesser | mzXML format data | “mass_dataset” class object | Raw data processing |
| ​​extract_eic() | massProcesser | - | - | Extract EIC |
| detect_outlier() | massCleaner | “mass_dataset” class object | “outlier_samples” class object | Detect outlier samples |
| extract_outlier_table() | massCleaner | “outlier_samples” class object | data.frame | Outlier sample table |
| impute_mv() | massCleaner | “mass_dataset” class object | “mass_dataset” class object | Impute missing values |
| integrate_data() | massCleaner | “mass_dataset” class object | “mass_dataset” class object | Integrate data |
| normalize_data() | massCleaner | “mass_dataset” class object | “mass_dataset” class object | Normalize data |
| optimize_loess_span() | massCleaner | “mass_dataset” class object | data.frame | Optimize the parameters for loess regression |
| align_batch() | massCleaner | “mass_dataset” class object | “mass_dataset” class object | Align two “mass_dataset” class objects |
| massqc_cumulative_rsd_plot() | massQC | “mass_dataset” class object | ggplot2 plot class object | Cummulative RSD plot |
| massqc_pca() | massQC | “mass_dataset” class object | ggplot2 plot class object | PCA score plot |
| massqc_rsd_plot() | massQC | “mass_dataset” class object | ggplot2 plot class object | RSD distribution plot |
| massqc_sample_boxplot() | massQC | “mass_dataset” class object | ggplot2 plot class object | Sample box plot |
| massqc_sample_correlation() | massQC | “mass_dataset” class object | ggplot2 plot class object | Sample correlation plot |
| massqc_report() | massQC | “mass_dataset” class object | HTML format report | HTML format QC report |
| annotate_metabolites_mass_dataset() | metID | “mass_dataset” class object | “mass_dataset” class object | Annotate features in “mass_dataset” by databases |
| annotate_single_peak_mass_dataset() | metID | “mass_dataset” class object | “mass_dataset” class object | Annotate one feature in “mass_dataset” by databases |
| ms2_plot_mass_dataset() | metID | “mass_dataset” class object | ggplot2 plot class object | MS^2^ matching plot |
| convert_dummy_variable() | massStat | vector | data.frame | Convert vector to dummy variable |
| convert_mass_dataset2graph() | massStat | “mass_dataset” class object | tbl_gh class | Convert “mass_dataset” class to graph object |
| cor_mass_dataset() | massStat | “mass_dataset” class object | data.frame | Correlation matrix |
| dist_mass_dataset() | massStat | “mass_dataset” class object | data.frame | Distance matrix |
| Heatmap() | massStat | ComplexHeatmap | ggplot2 plot class object | Heatmap plot |
| pls() | massStat | mixOmics | “pls” object | PLS analysis |
| plsda() | massStat | mixOmics | “plsda” object | PLS-DA analysis |
| mutate_fc() | massStat | “mass_dataset” class object | “mass_dataset” class object | Add fold change to variable information |
| mutate_p_value() | massStat | “mass_dataset” class object | “mass_dataset” class object | Univariable test |
| run_pca() | massStat | “mass_dataset” class object | pca class object | PCA analysis |
| pca_score_plot() | massStat | pca class object | ggplot2 plot class object | PCA score plot |
| scale_data() | massStat | “mass_dataset” class object | “mass_dataset” class object | Scale data |
| volcano_plot() | massStat | “mass_dataset” class | ggplot2 plot class object | Volcano plot |
| filter_pathway() | metPath | “pathway_database” class | “pathway_database” class | Filter pathways |
| get_hmdb_pathway() | metPath | - | “pathway_database” class | Get or download HMDB pathway |
| get_kegg_pathway() | metPath | - | “pathway_database” class | Get or download KEGG pathway |
| enrich_kegg() | metPath | Query metabolite ID | “enrich_result” class | Pathway enrichment |
| enrich_hmdb() | metPath | Query metabolite ID | “enrich_result” class | Pathway enrichment |
| enrich_bar_plot() | metPath | “enrich_result” class | ggplot2 plot class object | Barplot to show the enriched pathways |
| enrich_scatter_plot() | metPath | “enrich_result” class | ggplot2 plot class object | Scatter plot to show the enriched pathways |
| enrich_network() | metPath | “enrich_result” class | ggplot2 plot class object | Network to show the enriched pathways |
