# Supplementary material for: TidyMass an object-oriented reproducible analysis framework for LC–MS data
Source: Nat Commun. 2022 Jul 28;13:4365. doi: 10.1038/s41467-022-32155-w (PMC9334349; doi:10.1038/s41467-022-32155-w)
Supplement: Supplementary file 4 — Dataset 2 [file 41467_2022_32155_MOESM4_ESM.zip › Supplementary Data 2/statistical_analysis/parameter_report.html]

tidymass\_parameters.template.knit


# **Processing information**

### 2022-03-06

More information about this can be found here

---

| pacakge\_name | function\_name | parameter | time |
| --- | --- | --- | --- |
| massprocesser | process\_data | path:~/scratch60 | 2022-02-28 13:05:44 |
| massprocesser | process\_data | polarity:negative | 2022-02-28 13:05:44 |
| massprocesser | process\_data | ppm:20 | 2022-02-28 13:05:44 |
| massprocesser | process\_data | peakwidth:5,30 | 2022-02-28 13:05:44 |
| massprocesser | process\_data | snthresh:10 | 2022-02-28 13:05:44 |
| massprocesser | process\_data | prefilter:3,500 | 2022-02-28 13:05:44 |
| massprocesser | process\_data | fitgauss:FALSE | 2022-02-28 13:05:44 |
| massprocesser | process\_data | integrate:2 | 2022-02-28 13:05:44 |
| massprocesser | process\_data | mzdiff:0.01 | 2022-02-28 13:05:44 |
| massprocesser | process\_data | noise:500 | 2022-02-28 13:05:44 |
| massprocesser | process\_data | threads:6 | 2022-02-28 13:05:44 |
| massprocesser | process\_data | binSize:0.025 | 2022-02-28 13:05:44 |
| massprocesser | process\_data | bw:5 | 2022-02-28 13:05:44 |
| massprocesser | process\_data | output\_tic:FALSE | 2022-02-28 13:05:44 |
| massprocesser | process\_data | output\_bpc:FALSE | 2022-02-28 13:05:44 |
| massprocesser | process\_data | output\_rt\_correction\_plot:FALSE | 2022-02-28 13:05:44 |
| massprocesser | process\_data | min\_fraction:0.5 | 2022-02-28 13:05:44 |
| massprocesser | process\_data | fill\_peaks:FALSE | 2022-02-28 13:05:44 |
| massdataset | create\_mass\_dataset() | no:no | 2022-02-28 14:07:06 |
| massprocesser | process\_data | path:E:/2019\_CRC/20190109\_CRC\_RPLC50mm\_neg\_XCMS/tidymass | 2022-03-01 09:03:10 |
| massprocesser | process\_data | polarity:negative | 2022-03-01 09:03:10 |
| massprocesser | process\_data | ppm:20 | 2022-03-01 09:03:10 |
| massprocesser | process\_data | peakwidth:5,30 | 2022-03-01 09:03:10 |
| massprocesser | process\_data | snthresh:10 | 2022-03-01 09:03:10 |
| massprocesser | process\_data | prefilter:3,500 | 2022-03-01 09:03:10 |
| massprocesser | process\_data | fitgauss:FALSE | 2022-03-01 09:03:10 |
| massprocesser | process\_data | integrate:2 | 2022-03-01 09:03:10 |
| massprocesser | process\_data | mzdiff:0.01 | 2022-03-01 09:03:10 |
| massprocesser | process\_data | noise:500 | 2022-03-01 09:03:10 |
| massprocesser | process\_data | threads:6 | 2022-03-01 09:03:10 |
| massprocesser | process\_data | binSize:0.025 | 2022-03-01 09:03:10 |
| massprocesser | process\_data | bw:5 | 2022-03-01 09:03:10 |
| massprocesser | process\_data | output\_tic:FALSE | 2022-03-01 09:03:10 |
| massprocesser | process\_data | output\_bpc:FALSE | 2022-03-01 09:03:10 |
| massprocesser | process\_data | output\_rt\_correction\_plot:FALSE | 2022-03-01 09:03:10 |
| massprocesser | process\_data | min\_fraction:0.5 | 2022-03-01 09:03:10 |
| massprocesser | process\_data | fill\_peaks:FALSE | 2022-03-01 09:03:10 |
| massdataset | create\_mass\_dataset() | no:no | 2022-03-01 09:51:34 |
| massprocesser | process\_data | path:~/scratch60/CRC HILIC POS | 2022-03-02 07:55:29 |
| massprocesser | process\_data | polarity:positive | 2022-03-02 07:55:29 |
| massprocesser | process\_data | ppm:20 | 2022-03-02 07:55:29 |
| massprocesser | process\_data | peakwidth:5,30 | 2022-03-02 07:55:29 |
| massprocesser | process\_data | snthresh:10 | 2022-03-02 07:55:29 |
| massprocesser | process\_data | prefilter:3,500 | 2022-03-02 07:55:29 |
| massprocesser | process\_data | fitgauss:FALSE | 2022-03-02 07:55:29 |
| massprocesser | process\_data | integrate:2 | 2022-03-02 07:55:29 |
| massprocesser | process\_data | mzdiff:0.01 | 2022-03-02 07:55:29 |
| massprocesser | process\_data | noise:500 | 2022-03-02 07:55:29 |
| massprocesser | process\_data | threads:6 | 2022-03-02 07:55:29 |
| massprocesser | process\_data | binSize:0.025 | 2022-03-02 07:55:29 |
| massprocesser | process\_data | bw:5 | 2022-03-02 07:55:29 |
| massprocesser | process\_data | output\_tic:FALSE | 2022-03-02 07:55:29 |
| massprocesser | process\_data | output\_bpc:FALSE | 2022-03-02 07:55:29 |
| massprocesser | process\_data | output\_rt\_correction\_plot:FALSE | 2022-03-02 07:55:29 |
| massprocesser | process\_data | min\_fraction:0.5 | 2022-03-02 07:55:29 |
| massprocesser | process\_data | fill\_peaks:FALSE | 2022-03-02 07:55:29 |
| massdataset | create\_mass\_dataset() | no:no | 2022-03-02 09:06:33 |
| massprocesser | process\_data | path:~/scratch60/CRC RPLC POS | 2022-03-02 12:25:08 |
| massprocesser | process\_data | polarity:positive | 2022-03-02 12:25:08 |
| massprocesser | process\_data | ppm:20 | 2022-03-02 12:25:08 |
| massprocesser | process\_data | peakwidth:5,30 | 2022-03-02 12:25:08 |
| massprocesser | process\_data | snthresh:10 | 2022-03-02 12:25:08 |
| massprocesser | process\_data | prefilter:3,500 | 2022-03-02 12:25:08 |
| massprocesser | process\_data | fitgauss:FALSE | 2022-03-02 12:25:08 |
| massprocesser | process\_data | integrate:2 | 2022-03-02 12:25:08 |
| massprocesser | process\_data | mzdiff:0.01 | 2022-03-02 12:25:08 |
| massprocesser | process\_data | noise:500 | 2022-03-02 12:25:08 |
| massprocesser | process\_data | threads:6 | 2022-03-02 12:25:08 |
| massprocesser | process\_data | binSize:0.025 | 2022-03-02 12:25:08 |
| massprocesser | process\_data | bw:5 | 2022-03-02 12:25:08 |
| massprocesser | process\_data | output\_tic:FALSE | 2022-03-02 12:25:08 |
| massprocesser | process\_data | output\_bpc:FALSE | 2022-03-02 12:25:08 |
| massprocesser | process\_data | output\_rt\_correction\_plot:FALSE | 2022-03-02 12:25:08 |
| massprocesser | process\_data | min\_fraction:0.5 | 2022-03-02 12:25:08 |
| massprocesser | process\_data | fill\_peaks:FALSE | 2022-03-02 12:25:08 |
| massdataset | create\_mass\_dataset() | no:no | 2022-03-02 14:50:02 |
| massdataset | mutate() | parameter\_1:batch=as.character(batch) | 2022-03-05 16:52:56 |
| massdataset | mutate() | parameter\_1:batch=as.character(batch) | 2022-03-05 16:53:03 |
| massdataset | mutate() | parameter\_1:batch=as.character(batch) | 2022-03-05 16:53:24 |
| massdataset | mutate() | parameter\_1:batch=as.character(batch) | 2022-03-05 16:53:35 |
| massdataset | mutate\_variable\_na\_freq() | according\_to\_samples:QC01,QC02,QC03,QC04,QC05,… | 2022-03-05 17:01:27 |
| massdataset | mutate\_variable\_na\_freq() | according\_to\_samples:men\_normal\_166,men\_normal\_2332,men\_normal\_2357,men\_normal\_2371,men\_normal\_2407,… | 2022-03-05 17:01:27 |
| massdataset | mutate\_variable\_na\_freq() | according\_to\_samples:menLCCstage1\_1122,menLCCstage1\_1487,menLCCstage1\_1532,menLCCstage1\_1941,menLCCstage1\_1969,… | 2022-03-05 17:01:27 |
| massdataset | filter() | parameter:`~na_freq < 0.2 & (na_freq.1 < 0.5 &#124; na_freq.2 < 0.5)` | 2022-03-05 17:01:37 |
| massdataset | mutate\_variable\_na\_freq() | according\_to\_samples:QC01,QC02,QC03,QC04,QC05,… | 2022-03-05 17:04:03 |
| massdataset | mutate\_variable\_na\_freq() | according\_to\_samples:men\_normal\_166,men\_normal\_2332,men\_normal\_2357,men\_normal\_2371,men\_normal\_2407,… | 2022-03-05 17:04:03 |
| massdataset | mutate\_variable\_na\_freq() | according\_to\_samples:menLCCstage1\_1122,menLCCstage1\_1487,menLCCstage1\_1532,menLCCstage1\_1941,menLCCstage1\_1969,… | 2022-03-05 17:04:03 |
| massdataset | filter() | parameter:`~na_freq < 0.2 & (na_freq.1 < 0.5 &#124; na_freq.2 < 0.5)` | 2022-03-05 17:04:15 |
| massdataset | mutate\_variable\_na\_freq() | according\_to\_samples:QC01,QC02,QC03,QC04,QC05,… | 2022-03-05 17:06:07 |
| massdataset | mutate\_variable\_na\_freq() | according\_to\_samples:men\_normal\_166,men\_normal\_2332,men\_normal\_2357,men\_normal\_2371,men\_normal\_2407,… | 2022-03-05 17:06:07 |
| massdataset | mutate\_variable\_na\_freq() | according\_to\_samples:menLCCstage1\_1122,menLCCstage1\_1487,menLCCstage1\_1532,menLCCstage1\_1941,menLCCstage1\_1969,… | 2022-03-05 17:06:08 |
| massdataset | filter() | parameter:`~na_freq < 0.2 & (na_freq.1 < 0.5 &#124; na_freq.2 < 0.5)` | 2022-03-05 17:06:16 |
| massdataset | mutate\_variable\_na\_freq() | according\_to\_samples:QC01,QC02,QC03,QC04,QC05,… | 2022-03-05 17:08:15 |
| massdataset | mutate\_variable\_na\_freq() | according\_to\_samples:men\_normal\_166,men\_normal\_2332,men\_normal\_2357,men\_normal\_2371,men\_normal\_2407,… | 2022-03-05 17:08:15 |
| massdataset | mutate\_variable\_na\_freq() | according\_to\_samples:menLCCstage1\_1122,menLCCstage1\_1487,menLCCstage1\_1532,menLCCstage1\_1941,menLCCstage1\_1969,… | 2022-03-05 17:08:15 |
| massdataset | filter() | parameter:`~na_freq < 0.2 & (na_freq.1 < 0.5 &#124; na_freq.2 < 0.5)` | 2022-03-05 17:08:20 |
| massdataset | mutate() | parameter\_1:class=case\_when(class == “QC” ~ class, TRUE ~ “Subject”) | 2022-03-05 17:08:57 |
| massdataset | mutate() | parameter\_1:class=case\_when(class == “QC” ~ class, TRUE ~ “Subject”) | 2022-03-05 17:10:13 |
| massdataset | mutate() | parameter\_1:class=case\_when(class == “QC” ~ class, TRUE ~ “Subject”) | 2022-03-05 17:18:51 |
| massdataset | mutate() | parameter\_1:class=case\_when(class == “QC” ~ class, TRUE ~ “Subject”) | 2022-03-05 17:19:31 |
| masscleaner | impute\_mv() | method:knn | 2022-03-05 17:45:56 |
| masscleaner | impute\_mv() | rowmax:0.5 | 2022-03-05 17:45:56 |
| masscleaner | impute\_mv() | colmax:0.9 | 2022-03-05 17:45:56 |
| masscleaner | impute\_mv() | maxp:1500 | 2022-03-05 17:45:56 |
| masscleaner | impute\_mv() | rng.seed:362436069 | 2022-03-05 17:45:56 |
| masscleaner | impute\_mv() | sample\_id:men\_normal\_166,men\_normal\_2332,men\_normal\_2357,men\_normal\_2371,men\_normal\_2407,… | 2022-03-05 17:45:56 |
| masscleaner | impute\_mv() | method:knn | 2022-03-05 17:46:18 |
| masscleaner | impute\_mv() | rowmax:0.5 | 2022-03-05 17:46:18 |
| masscleaner | impute\_mv() | colmax:0.8 | 2022-03-05 17:46:18 |
| masscleaner | impute\_mv() | maxp:1500 | 2022-03-05 17:46:18 |
| masscleaner | impute\_mv() | rng.seed:362436069 | 2022-03-05 17:46:18 |
| masscleaner | impute\_mv() | sample\_id:men\_normal\_166,men\_normal\_2332,men\_normal\_2357,men\_normal\_2371,men\_normal\_2407,… | 2022-03-05 17:46:18 |
| masscleaner | impute\_mv() | method:knn | 2022-03-05 17:46:40 |
| masscleaner | impute\_mv() | rowmax:0.5 | 2022-03-05 17:46:40 |
| masscleaner | impute\_mv() | colmax:0.8 | 2022-03-05 17:46:40 |
| masscleaner | impute\_mv() | maxp:1500 | 2022-03-05 17:46:40 |
| masscleaner | impute\_mv() | rng.seed:362436069 | 2022-03-05 17:46:40 |
| masscleaner | impute\_mv() | sample\_id:men\_normal\_166,men\_normal\_2332,men\_normal\_2357,men\_normal\_2371,men\_normal\_2407,… | 2022-03-05 17:46:40 |
| masscleaner | impute\_mv() | method:knn | 2022-03-05 17:46:54 |
| masscleaner | impute\_mv() | rowmax:0.5 | 2022-03-05 17:46:54 |
| masscleaner | impute\_mv() | colmax:0.8 | 2022-03-05 17:46:54 |
| masscleaner | impute\_mv() | maxp:1500 | 2022-03-05 17:46:54 |
| masscleaner | impute\_mv() | rng.seed:362436069 | 2022-03-05 17:46:54 |
| masscleaner | impute\_mv() | sample\_id:menLCCstage1\_1122,menLCCstage1\_1487,menLCCstage1\_1532,menLCCstage1\_1941,menLCCstage1\_1969,… | 2022-03-05 17:46:54 |
| masscleaner | normalize\_data() | method:svr | 2022-03-05 19:18:27 |
| masscleaner | normalize\_data() | keep\_scale:TRUE | 2022-03-05 19:18:27 |
| masscleaner | normalize\_data() | multiple:1 | 2022-03-05 19:18:27 |
| masscleaner | normalize\_data() | threads:4 | 2022-03-05 19:18:27 |
| masscleaner | normalize\_data() | method:svr | 2022-03-05 19:56:37 |
| masscleaner | normalize\_data() | keep\_scale:TRUE | 2022-03-05 19:56:37 |
| masscleaner | normalize\_data() | multiple:1 | 2022-03-05 19:56:37 |
| masscleaner | normalize\_data() | threads:4 | 2022-03-05 19:56:37 |
| masscleaner | normalize\_data() | method:svr | 2022-03-05 20:54:20 |
| masscleaner | normalize\_data() | keep\_scale:TRUE | 2022-03-05 20:54:20 |
| masscleaner | normalize\_data() | multiple:1 | 2022-03-05 20:54:20 |
| masscleaner | normalize\_data() | threads:4 | 2022-03-05 20:54:20 |
| masscleaner | normalize\_data() | method:svr | 2022-03-05 21:06:01 |
| masscleaner | normalize\_data() | keep\_scale:TRUE | 2022-03-05 21:06:01 |
| masscleaner | normalize\_data() | multiple:1 | 2022-03-05 21:06:01 |
| masscleaner | normalize\_data() | threads:4 | 2022-03-05 21:06:01 |
| metid | annotate\_metabolites\_mass\_dataset() | ms1.match.ppm:15 | 2022-03-05 23:36:53 |
| metid | annotate\_metabolites\_mass\_dataset() | ms2.match.ppm:30 | 2022-03-05 23:36:53 |
| metid | annotate\_metabolites\_mass\_dataset() | mz.ppm.thr:400 | 2022-03-05 23:36:53 |
| metid | annotate\_metabolites\_mass\_dataset() | ms2.match.tol:0.5 | 2022-03-05 23:36:53 |
| metid | annotate\_metabolites\_mass\_dataset() | fraction.weight:0.3 | 2022-03-05 23:36:53 |
| metid | annotate\_metabolites\_mass\_dataset() | dp.forward.weight:0.6 | 2022-03-05 23:36:53 |
| metid | annotate\_metabolites\_mass\_dataset() | dp.reverse.weight:0.1 | 2022-03-05 23:36:53 |
| metid | annotate\_metabolites\_mass\_dataset() | rt.match.tol:30 | 2022-03-05 23:36:53 |
| metid | annotate\_metabolites\_mass\_dataset() | polarity:positive | 2022-03-05 23:36:53 |
| metid | annotate\_metabolites\_mass\_dataset() | ce:all | 2022-03-05 23:36:53 |
| metid | annotate\_metabolites\_mass\_dataset() | column:rp | 2022-03-05 23:36:53 |
| metid | annotate\_metabolites\_mass\_dataset() | ms1.match.weight:0.25 | 2022-03-05 23:36:53 |
| metid | annotate\_metabolites\_mass\_dataset() | rt.match.weight:0.25 | 2022-03-05 23:36:53 |
| metid | annotate\_metabolites\_mass\_dataset() | ms2.match.weight:0.5 | 2022-03-05 23:36:53 |
| metid | annotate\_metabolites\_mass\_dataset() | total.score.tol:0.5 | 2022-03-05 23:36:53 |
| metid | annotate\_metabolites\_mass\_dataset() | candidate.num:3 | 2022-03-05 23:36:53 |
| metid | annotate\_metabolites\_mass\_dataset() | database:Michael Snyder lab\_0.0.1 | 2022-03-05 23:36:53 |
| metid | annotate\_metabolites\_mass\_dataset() | threads:3 | 2022-03-05 23:36:53 |
| metid | annotate\_metabolites\_mass\_dataset() | ms1.match.ppm:15 | 2022-03-06 00:13:48 |
| metid | annotate\_metabolites\_mass\_dataset() | ms2.match.ppm:30 | 2022-03-06 00:13:48 |
| metid | annotate\_metabolites\_mass\_dataset() | mz.ppm.thr:400 | 2022-03-06 00:13:48 |
| metid | annotate\_metabolites\_mass\_dataset() | ms2.match.tol:0.5 | 2022-03-06 00:13:48 |
| metid | annotate\_metabolites\_mass\_dataset() | fraction.weight:0.3 | 2022-03-06 00:13:48 |
| metid | annotate\_metabolites\_mass\_dataset() | dp.forward.weight:0.6 | 2022-03-06 00:13:48 |
| metid | annotate\_metabolites\_mass\_dataset() | dp.reverse.weight:0.1 | 2022-03-06 00:13:48 |
| metid | annotate\_metabolites\_mass\_dataset() | rt.match.tol:1e+06 | 2022-03-06 00:13:48 |
| metid | annotate\_metabolites\_mass\_dataset() | polarity:positive | 2022-03-06 00:13:48 |
| metid | annotate\_metabolites\_mass\_dataset() | ce:all | 2022-03-06 00:13:48 |
| metid | annotate\_metabolites\_mass\_dataset() | column:rp | 2022-03-06 00:13:48 |
| metid | annotate\_metabolites\_mass\_dataset() | ms1.match.weight:0.25 | 2022-03-06 00:13:48 |
| metid | annotate\_metabolites\_mass\_dataset() | rt.match.weight:0.25 | 2022-03-06 00:13:48 |
| metid | annotate\_metabolites\_mass\_dataset() | ms2.match.weight:0.5 | 2022-03-06 00:13:48 |
| metid | annotate\_metabolites\_mass\_dataset() | total.score.tol:0.5 | 2022-03-06 00:13:48 |
| metid | annotate\_metabolites\_mass\_dataset() | candidate.num:3 | 2022-03-06 00:13:48 |
| metid | annotate\_metabolites\_mass\_dataset() | database:MS\_0.0.2 | 2022-03-06 00:13:48 |
| metid | annotate\_metabolites\_mass\_dataset() | threads:5 | 2022-03-06 00:13:48 |
| metid | annotate\_metabolites\_mass\_dataset() | ms1.match.ppm:15 | 2022-03-06 00:29:13 |
| metid | annotate\_metabolites\_mass\_dataset() | ms2.match.ppm:30 | 2022-03-06 00:29:13 |
| metid | annotate\_metabolites\_mass\_dataset() | mz.ppm.thr:400 | 2022-03-06 00:29:13 |
| metid | annotate\_metabolites\_mass\_dataset() | ms2.match.tol:0.5 | 2022-03-06 00:29:13 |
| metid | annotate\_metabolites\_mass\_dataset() | fraction.weight:0.3 | 2022-03-06 00:29:13 |
| metid | annotate\_metabolites\_mass\_dataset() | dp.forward.weight:0.6 | 2022-03-06 00:29:13 |
| metid | annotate\_metabolites\_mass\_dataset() | dp.reverse.weight:0.1 | 2022-03-06 00:29:13 |
| metid | annotate\_metabolites\_mass\_dataset() | rt.match.tol:30 | 2022-03-06 00:29:13 |
| metid | annotate\_metabolites\_mass\_dataset() | polarity:negative | 2022-03-06 00:29:13 |
| metid | annotate\_metabolites\_mass\_dataset() | ce:all | 2022-03-06 00:29:13 |
| metid | annotate\_metabolites\_mass\_dataset() | column:rp | 2022-03-06 00:29:13 |
| metid | annotate\_metabolites\_mass\_dataset() | ms1.match.weight:0.25 | 2022-03-06 00:29:13 |
| metid | annotate\_metabolites\_mass\_dataset() | rt.match.weight:0.25 | 2022-03-06 00:29:13 |
| metid | annotate\_metabolites\_mass\_dataset() | ms2.match.weight:0.5 | 2022-03-06 00:29:13 |
| metid | annotate\_metabolites\_mass\_dataset() | total.score.tol:0.5 | 2022-03-06 00:29:13 |
| metid | annotate\_metabolites\_mass\_dataset() | candidate.num:3 | 2022-03-06 00:29:13 |
| metid | annotate\_metabolites\_mass\_dataset() | database:Michael Snyder lab\_0.0.1 | 2022-03-06 00:29:13 |
| metid | annotate\_metabolites\_mass\_dataset() | threads:5 | 2022-03-06 00:29:13 |
| metid | annotate\_metabolites\_mass\_dataset() | ms1.match.ppm:15 | 2022-03-06 00:36:58 |
| metid | annotate\_metabolites\_mass\_dataset() | ms2.match.ppm:30 | 2022-03-06 00:36:58 |
| metid | annotate\_metabolites\_mass\_dataset() | mz.ppm.thr:400 | 2022-03-06 00:36:58 |
| metid | annotate\_metabolites\_mass\_dataset() | ms2.match.tol:0.5 | 2022-03-06 00:36:58 |
| metid | annotate\_metabolites\_mass\_dataset() | fraction.weight:0.3 | 2022-03-06 00:36:58 |
| metid | annotate\_metabolites\_mass\_dataset() | dp.forward.weight:0.6 | 2022-03-06 00:36:58 |
| metid | annotate\_metabolites\_mass\_dataset() | dp.reverse.weight:0.1 | 2022-03-06 00:36:58 |
| metid | annotate\_metabolites\_mass\_dataset() | rt.match.tol:1e+06 | 2022-03-06 00:36:58 |
| metid | annotate\_metabolites\_mass\_dataset() | polarity:negative | 2022-03-06 00:36:58 |
| metid | annotate\_metabolites\_mass\_dataset() | ce:all | 2022-03-06 00:36:58 |
| metid | annotate\_metabolites\_mass\_dataset() | column:rp | 2022-03-06 00:36:58 |
| metid | annotate\_metabolites\_mass\_dataset() | ms1.match.weight:0.25 | 2022-03-06 00:36:58 |
| metid | annotate\_metabolites\_mass\_dataset() | rt.match.weight:0.25 | 2022-03-06 00:36:58 |
| metid | annotate\_metabolites\_mass\_dataset() | ms2.match.weight:0.5 | 2022-03-06 00:36:58 |
| metid | annotate\_metabolites\_mass\_dataset() | total.score.tol:0.5 | 2022-03-06 00:36:58 |
| metid | annotate\_metabolites\_mass\_dataset() | candidate.num:3 | 2022-03-06 00:36:58 |
| metid | annotate\_metabolites\_mass\_dataset() | database:MS\_0.0.2 | 2022-03-06 00:36:58 |
| metid | annotate\_metabolites\_mass\_dataset() | threads:5 | 2022-03-06 00:36:58 |
| metid | annotate\_metabolites\_mass\_dataset() | ms1.match.ppm:15 | 2022-03-06 00:43:12 |
| metid | annotate\_metabolites\_mass\_dataset() | ms2.match.ppm:30 | 2022-03-06 00:43:12 |
| metid | annotate\_metabolites\_mass\_dataset() | mz.ppm.thr:400 | 2022-03-06 00:43:12 |
| metid | annotate\_metabolites\_mass\_dataset() | ms2.match.tol:0.5 | 2022-03-06 00:43:12 |
| metid | annotate\_metabolites\_mass\_dataset() | fraction.weight:0.3 | 2022-03-06 00:43:12 |
| metid | annotate\_metabolites\_mass\_dataset() | dp.forward.weight:0.6 | 2022-03-06 00:43:12 |
| metid | annotate\_metabolites\_mass\_dataset() | dp.reverse.weight:0.1 | 2022-03-06 00:43:12 |
| metid | annotate\_metabolites\_mass\_dataset() | rt.match.tol:30 | 2022-03-06 00:43:12 |
| metid | annotate\_metabolites\_mass\_dataset() | polarity:positive | 2022-03-06 00:43:12 |
| metid | annotate\_metabolites\_mass\_dataset() | ce:all | 2022-03-06 00:43:12 |
| metid | annotate\_metabolites\_mass\_dataset() | column:rp | 2022-03-06 00:43:12 |
| metid | annotate\_metabolites\_mass\_dataset() | ms1.match.weight:0.25 | 2022-03-06 00:43:12 |
| metid | annotate\_metabolites\_mass\_dataset() | rt.match.weight:0.25 | 2022-03-06 00:43:12 |
| metid | annotate\_metabolites\_mass\_dataset() | ms2.match.weight:0.5 | 2022-03-06 00:43:12 |
| metid | annotate\_metabolites\_mass\_dataset() | total.score.tol:0.5 | 2022-03-06 00:43:12 |
| metid | annotate\_metabolites\_mass\_dataset() | candidate.num:3 | 2022-03-06 00:43:12 |
| metid | annotate\_metabolites\_mass\_dataset() | database:Michael Snyder lab\_0.0.1 | 2022-03-06 00:43:12 |
| metid | annotate\_metabolites\_mass\_dataset() | threads:5 | 2022-03-06 00:43:12 |
| metid | annotate\_metabolites\_mass\_dataset() | ms1.match.ppm:15 | 2022-03-06 00:48:51 |
| metid | annotate\_metabolites\_mass\_dataset() | ms2.match.ppm:30 | 2022-03-06 00:48:51 |
| metid | annotate\_metabolites\_mass\_dataset() | mz.ppm.thr:400 | 2022-03-06 00:48:51 |
| metid | annotate\_metabolites\_mass\_dataset() | ms2.match.tol:0.5 | 2022-03-06 00:48:51 |
| metid | annotate\_metabolites\_mass\_dataset() | fraction.weight:0.3 | 2022-03-06 00:48:51 |
| metid | annotate\_metabolites\_mass\_dataset() | dp.forward.weight:0.6 | 2022-03-06 00:48:51 |
| metid | annotate\_metabolites\_mass\_dataset() | dp.reverse.weight:0.1 | 2022-03-06 00:48:51 |
| metid | annotate\_metabolites\_mass\_dataset() | rt.match.tol:1e+06 | 2022-03-06 00:48:51 |
| metid | annotate\_metabolites\_mass\_dataset() | polarity:positive | 2022-03-06 00:48:51 |
| metid | annotate\_metabolites\_mass\_dataset() | ce:all | 2022-03-06 00:48:51 |
| metid | annotate\_metabolites\_mass\_dataset() | column:rp | 2022-03-06 00:48:51 |
| metid | annotate\_metabolites\_mass\_dataset() | ms1.match.weight:0.25 | 2022-03-06 00:48:51 |
| metid | annotate\_metabolites\_mass\_dataset() | rt.match.weight:0.25 | 2022-03-06 00:48:51 |
| metid | annotate\_metabolites\_mass\_dataset() | ms2.match.weight:0.5 | 2022-03-06 00:48:51 |
| metid | annotate\_metabolites\_mass\_dataset() | total.score.tol:0.5 | 2022-03-06 00:48:51 |
| metid | annotate\_metabolites\_mass\_dataset() | candidate.num:3 | 2022-03-06 00:48:51 |
| metid | annotate\_metabolites\_mass\_dataset() | database:MS\_0.0.2 | 2022-03-06 00:48:51 |
| metid | annotate\_metabolites\_mass\_dataset() | threads:5 | 2022-03-06 00:48:51 |
| metid | annotate\_metabolites\_mass\_dataset() | ms1.match.ppm:15 | 2022-03-06 00:58:51 |
| metid | annotate\_metabolites\_mass\_dataset() | ms2.match.ppm:30 | 2022-03-06 00:58:51 |
| metid | annotate\_metabolites\_mass\_dataset() | mz.ppm.thr:400 | 2022-03-06 00:58:51 |
| metid | annotate\_metabolites\_mass\_dataset() | ms2.match.tol:0.5 | 2022-03-06 00:58:51 |
| metid | annotate\_metabolites\_mass\_dataset() | fraction.weight:0.3 | 2022-03-06 00:58:51 |
| metid | annotate\_metabolites\_mass\_dataset() | dp.forward.weight:0.6 | 2022-03-06 00:58:51 |
| metid | annotate\_metabolites\_mass\_dataset() | dp.reverse.weight:0.1 | 2022-03-06 00:58:51 |
| metid | annotate\_metabolites\_mass\_dataset() | rt.match.tol:30 | 2022-03-06 00:58:51 |
| metid | annotate\_metabolites\_mass\_dataset() | polarity:negative | 2022-03-06 00:58:51 |
| metid | annotate\_metabolites\_mass\_dataset() | ce:all | 2022-03-06 00:58:51 |
| metid | annotate\_metabolites\_mass\_dataset() | column:rp | 2022-03-06 00:58:51 |
| metid | annotate\_metabolites\_mass\_dataset() | ms1.match.weight:0.25 | 2022-03-06 00:58:51 |
| metid | annotate\_metabolites\_mass\_dataset() | rt.match.weight:0.25 | 2022-03-06 00:58:51 |
| metid | annotate\_metabolites\_mass\_dataset() | ms2.match.weight:0.5 | 2022-03-06 00:58:51 |
| metid | annotate\_metabolites\_mass\_dataset() | total.score.tol:0.5 | 2022-03-06 00:58:51 |
| metid | annotate\_metabolites\_mass\_dataset() | candidate.num:3 | 2022-03-06 00:58:51 |
| metid | annotate\_metabolites\_mass\_dataset() | database:Michael Snyder lab\_0.0.1 | 2022-03-06 00:58:51 |
| metid | annotate\_metabolites\_mass\_dataset() | threads:5 | 2022-03-06 00:58:51 |
| metid | annotate\_metabolites\_mass\_dataset() | ms1.match.ppm:15 | 2022-03-06 01:14:54 |
| metid | annotate\_metabolites\_mass\_dataset() | ms2.match.ppm:30 | 2022-03-06 01:14:54 |
| metid | annotate\_metabolites\_mass\_dataset() | mz.ppm.thr:400 | 2022-03-06 01:14:54 |
| metid | annotate\_metabolites\_mass\_dataset() | ms2.match.tol:0.5 | 2022-03-06 01:14:54 |
| metid | annotate\_metabolites\_mass\_dataset() | fraction.weight:0.3 | 2022-03-06 01:14:54 |
| metid | annotate\_metabolites\_mass\_dataset() | dp.forward.weight:0.6 | 2022-03-06 01:14:54 |
| metid | annotate\_metabolites\_mass\_dataset() | dp.reverse.weight:0.1 | 2022-03-06 01:14:54 |
| metid | annotate\_metabolites\_mass\_dataset() | rt.match.tol:1e+06 | 2022-03-06 01:14:54 |
| metid | annotate\_metabolites\_mass\_dataset() | polarity:negative | 2022-03-06 01:14:54 |
| metid | annotate\_metabolites\_mass\_dataset() | ce:all | 2022-03-06 01:14:54 |
| metid | annotate\_metabolites\_mass\_dataset() | column:rp | 2022-03-06 01:14:54 |
| metid | annotate\_metabolites\_mass\_dataset() | ms1.match.weight:0.25 | 2022-03-06 01:14:54 |
| metid | annotate\_metabolites\_mass\_dataset() | rt.match.weight:0.25 | 2022-03-06 01:14:54 |
| metid | annotate\_metabolites\_mass\_dataset() | ms2.match.weight:0.5 | 2022-03-06 01:14:54 |
| metid | annotate\_metabolites\_mass\_dataset() | total.score.tol:0.5 | 2022-03-06 01:14:54 |
| metid | annotate\_metabolites\_mass\_dataset() | candidate.num:3 | 2022-03-06 01:14:54 |
| metid | annotate\_metabolites\_mass\_dataset() | database:MS\_0.0.2 | 2022-03-06 01:14:54 |
| metid | annotate\_metabolites\_mass\_dataset() | threads:5 | 2022-03-06 01:14:54 |
| massdataset | filter() | parameter:`~!is.na(Level)` | 2022-03-06 01:16:20 |
| massdataset | filter() | parameter:`~Level == 1 &#124; Level == 2` | 2022-03-06 01:16:20 |
| massdataset | filter() | parameter:`~!is.na(Level)` | 2022-03-06 01:16:23 |
| massdataset | filter() | parameter:`~Level == 1 &#124; Level == 2` | 2022-03-06 01:16:23 |
| massdataset | filter() | parameter:`~!is.na(Level)` | 2022-03-06 01:16:25 |
| massdataset | filter() | parameter:`~Level == 1 &#124; Level == 2` | 2022-03-06 01:16:25 |
| massdataset | filter() | parameter:`~!is.na(Level)` | 2022-03-06 01:16:27 |
| massdataset | filter() | parameter:`~Level == 1 &#124; Level == 2` | 2022-03-06 01:16:27 |
| massdataset | merge\_mass\_dataset | sample\_direction:inner | 2022-03-06 01:19:43 |
| massdataset | merge\_mass\_dataset | variable\_direction:full | 2022-03-06 01:19:43 |
| massdataset | merge\_mass\_dataset | sample\_by:sample\_id | 2022-03-06 01:19:43 |
| massdataset | merge\_mass\_dataset | variable\_by:variable\_id,mz,rt | 2022-03-06 01:19:43 |
| massdataset | merge\_mass\_dataset | sample\_direction:inner | 2022-03-06 01:20:04 |
| massdataset | merge\_mass\_dataset | variable\_direction:full | 2022-03-06 01:20:04 |
| massdataset | merge\_mass\_dataset | sample\_by:sample\_id | 2022-03-06 01:20:04 |
| massdataset | merge\_mass\_dataset | variable\_by:variable\_id,mz,rt | 2022-03-06 01:20:04 |
| massdataset | merge\_mass\_dataset | sample\_direction:inner | 2022-03-06 01:24:10 |
| massdataset | merge\_mass\_dataset | variable\_direction:full | 2022-03-06 01:24:10 |
| massdataset | merge\_mass\_dataset | sample\_by:sample\_id | 2022-03-06 01:24:10 |
| massdataset | merge\_mass\_dataset | variable\_by:variable\_id,mz,rt | 2022-03-06 01:24:10 |
| massdataset | filter() | parameter:`~Level == min(Level)` | 2022-03-06 01:25:50 |
| massdataset | filter() | parameter:`~Total.score == max(Total.score)` | 2022-03-06 01:25:50 |
| massdataset | slice\_head() | n:1 | 2022-03-06 01:25:50 |
| massdataset | slice\_head() | prop:missing | 2022-03-06 01:25:50 |
