# Supplementary figures and images for: TidyMass an object-oriented reproducible analysis framework for LC–MS data
Source: Nat Commun. 2022 Jul 28;13:4365. doi: 10.1038/s41467-022-32155-w (PMC9334349; doi:10.1038/s41467-022-32155-w)

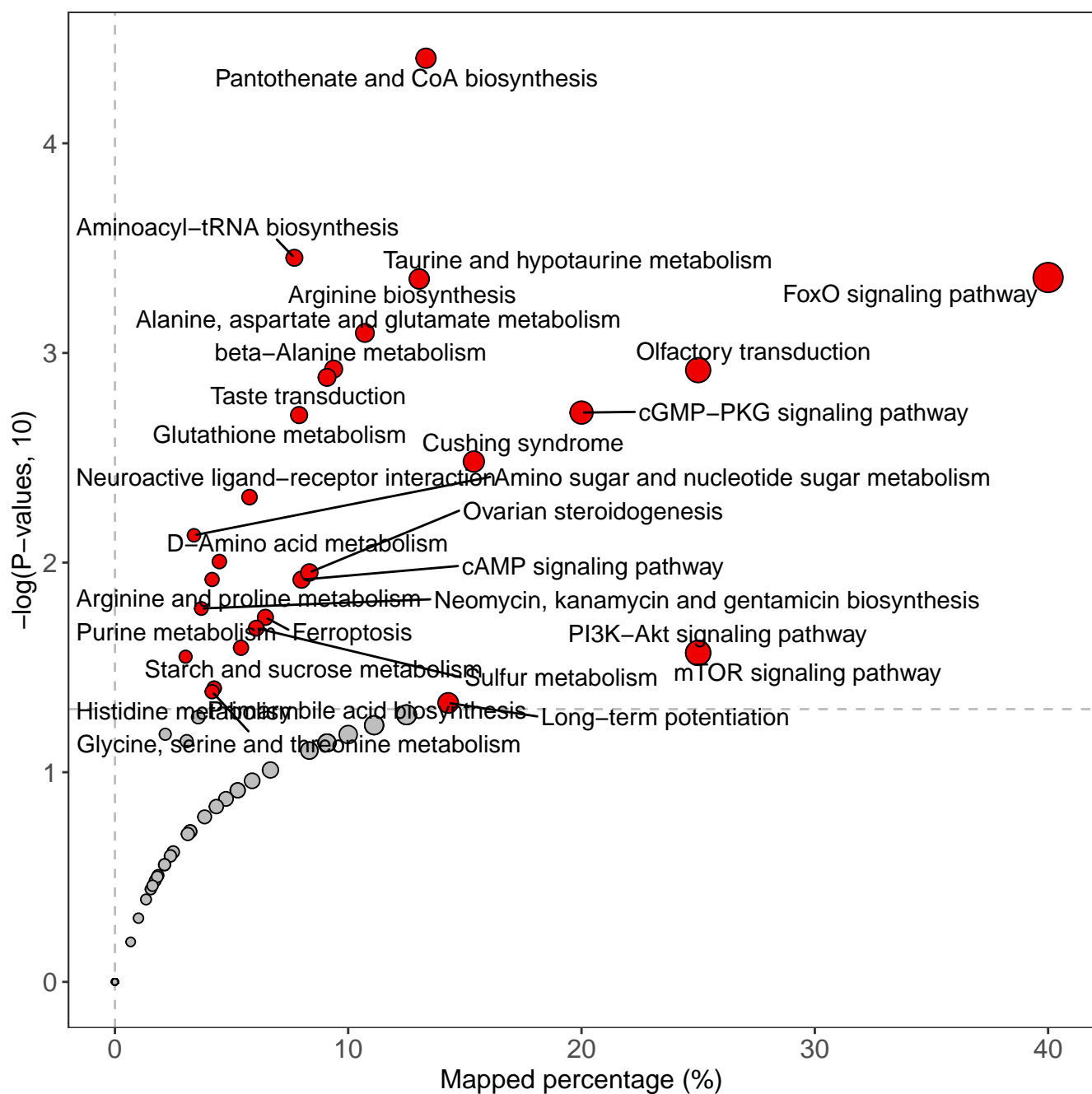

Supplement: Supplementary file 4 — Dataset 2 [file 41467_2022_32155_MOESM4_ESM.zip › Supplementary Data 2/pathway_enrichment/male_pathway_scatter.pdf]

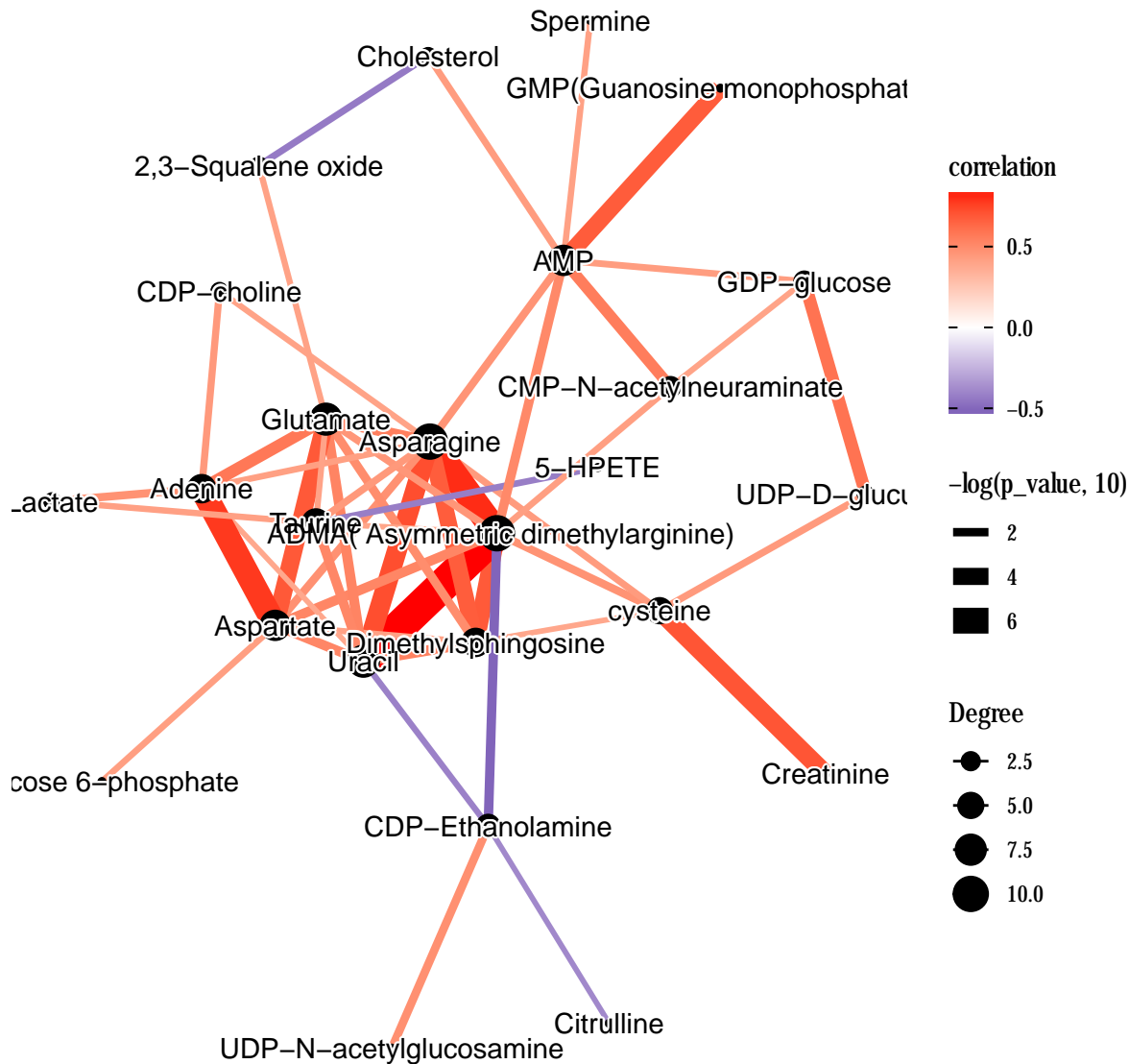

Supplement: Supplementary file 4 — Dataset 2 [file 41467_2022_32155_MOESM4_ESM.zip › Supplementary Data 2/pathway_enrichment/male_cor_network.pdf]

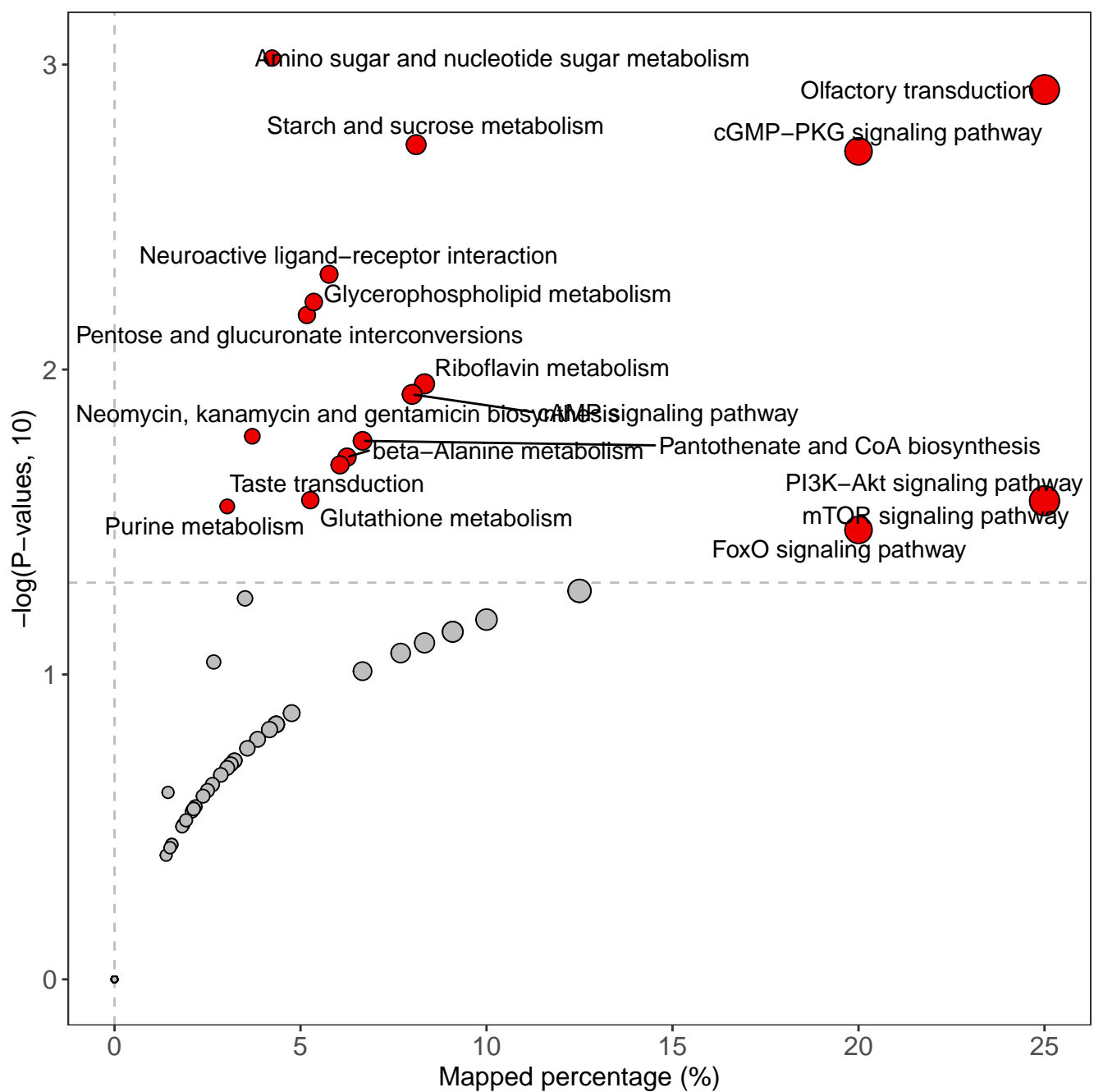

Supplement: Supplementary file 4 — Dataset 2 [file 41467_2022_32155_MOESM4_ESM.zip › Supplementary Data 2/pathway_enrichment/female_pathway_scatter.pdf]

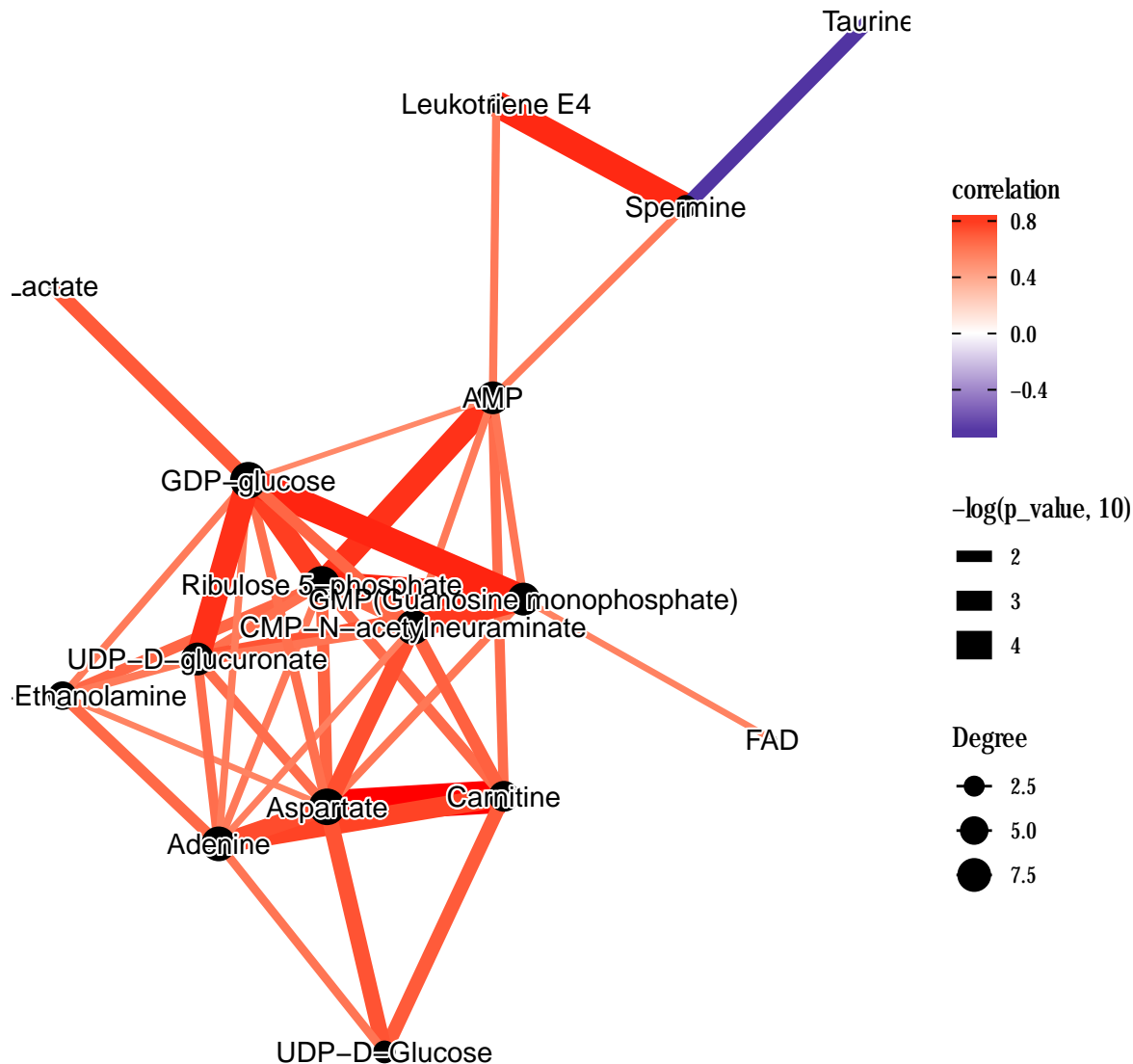

Supplement: Supplementary file 4 — Dataset 2 [file 41467_2022_32155_MOESM4_ESM.zip › Supplementary Data 2/pathway_enrichment/female_cor_network.pdf]

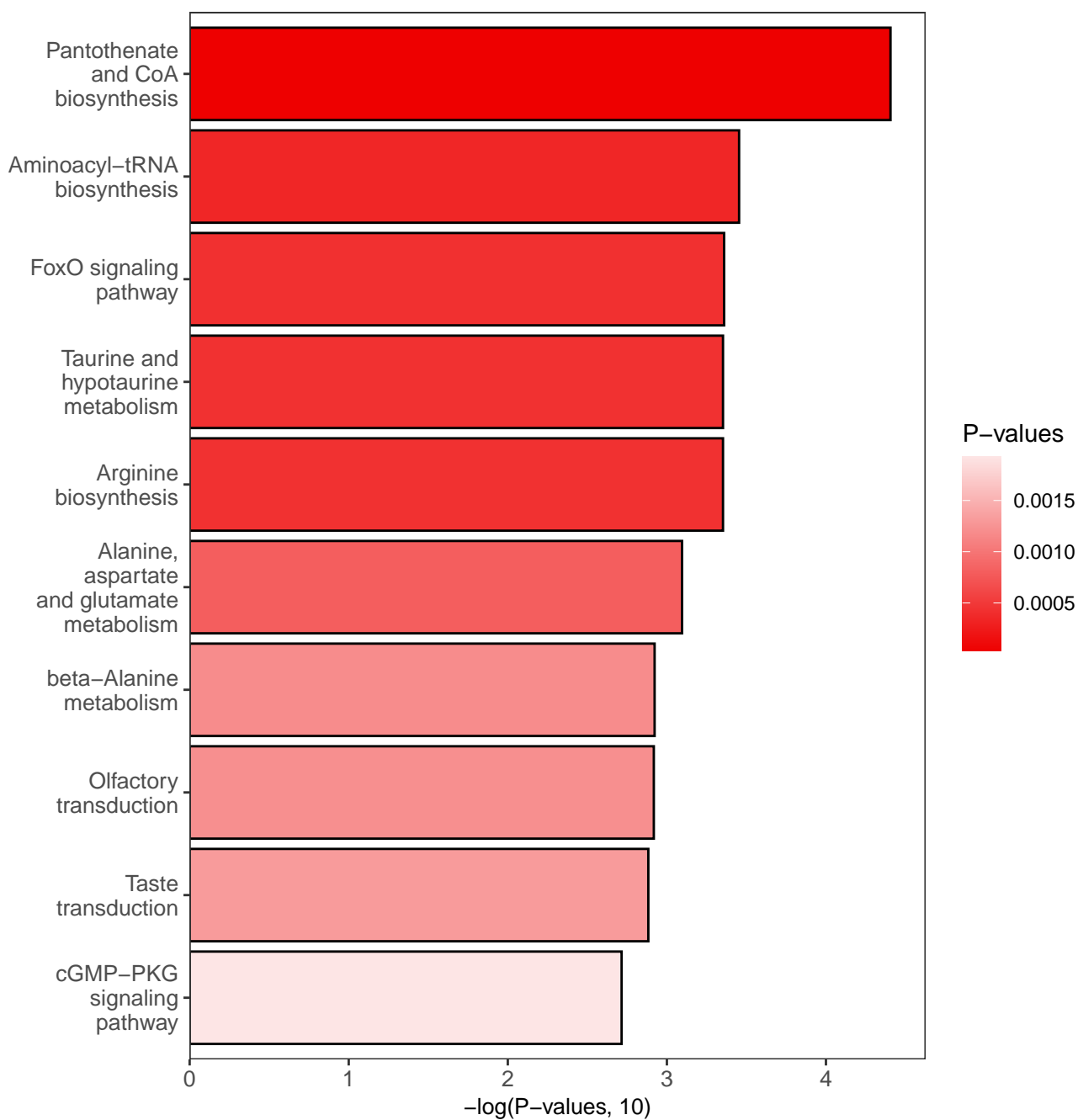

Supplement: Supplementary file 4 — Dataset 2 [file 41467_2022_32155_MOESM4_ESM.zip › Supplementary Data 2/pathway_enrichment/male_pathway_bar.pdf]

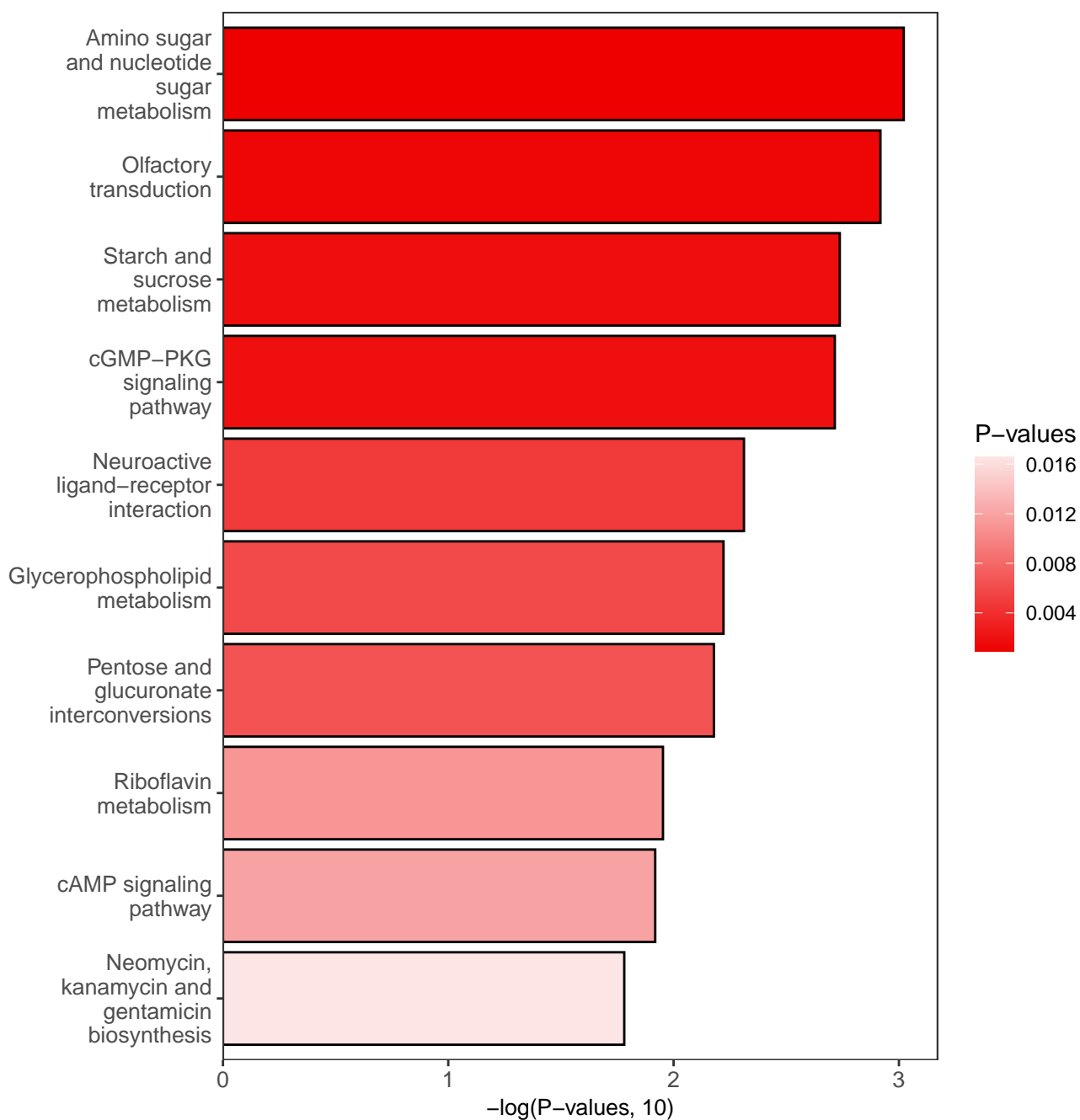

Supplement: Supplementary file 4 — Dataset 2 [file 41467_2022_32155_MOESM4_ESM.zip › Supplementary Data 2/pathway_enrichment/female_pathway_bar.pdf]

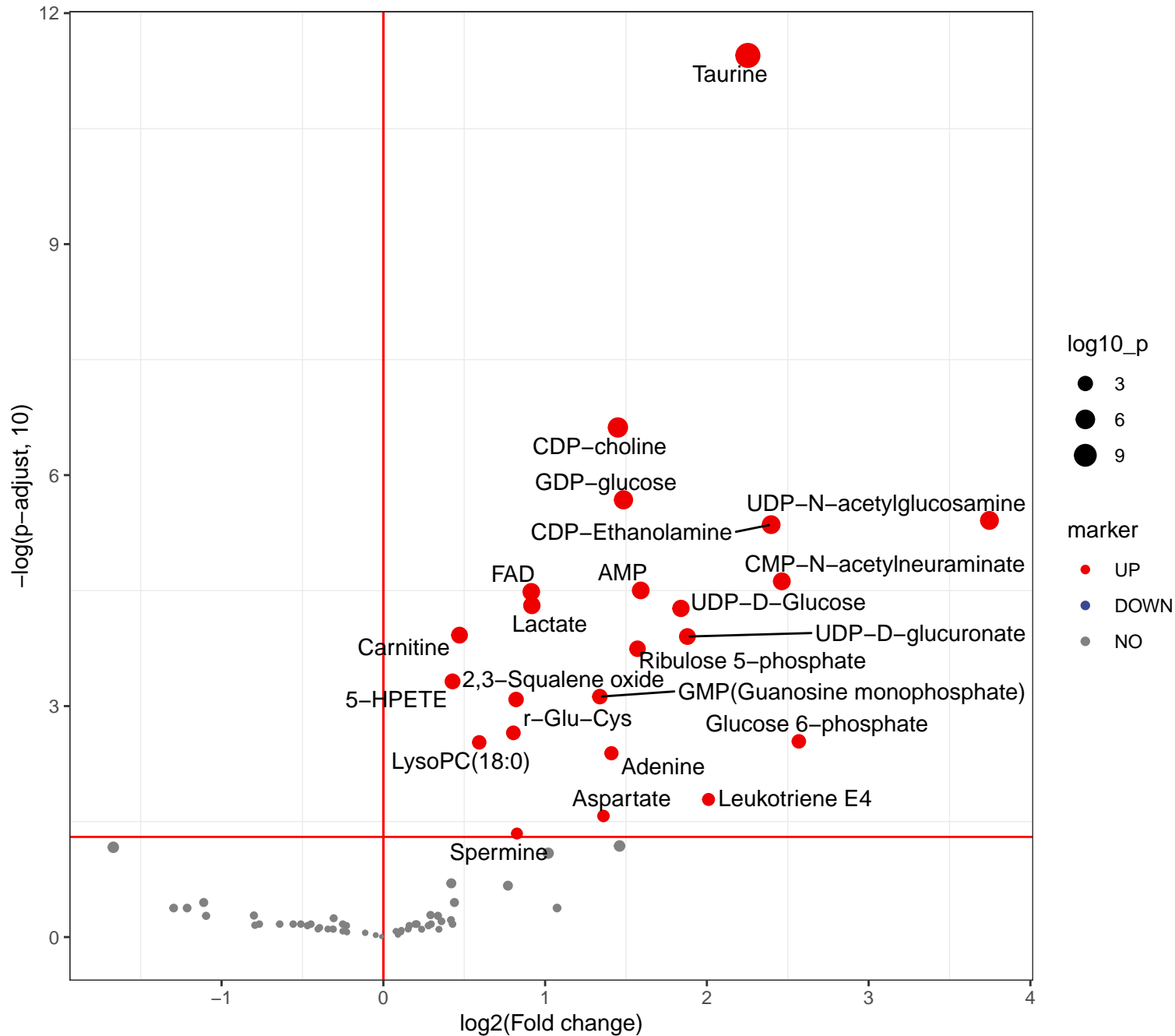

Supplement: Supplementary file 4 — Dataset 2 [file 41467_2022_32155_MOESM4_ESM.zip › Supplementary Data 2/statistical_analysis/female_LCC_vs_femal_control.pdf]

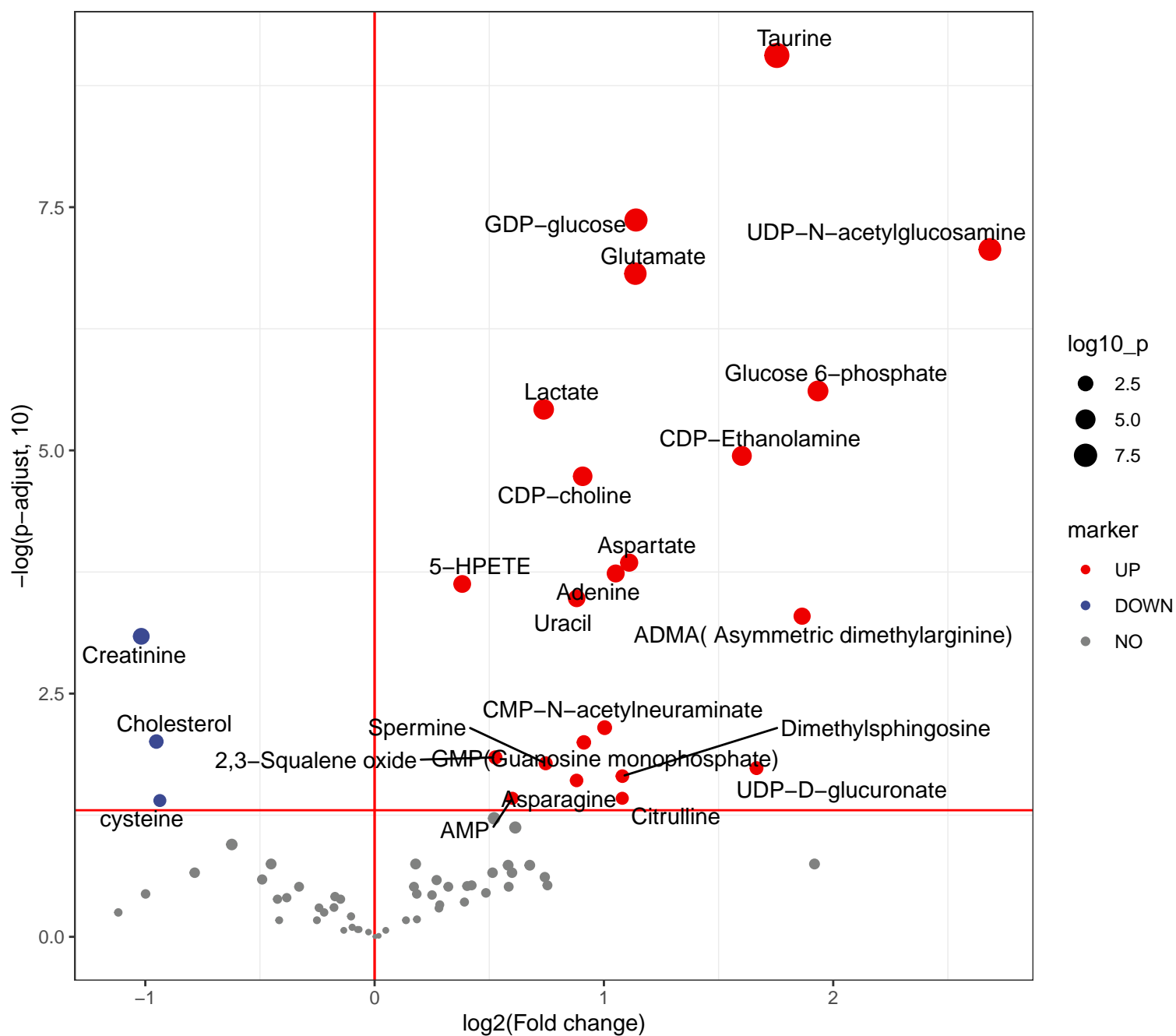

Supplement: Supplementary file 4 — Dataset 2 [file 41467_2022_32155_MOESM4_ESM.zip › Supplementary Data 2/statistical_analysis/male_LCC_vs_male_control.pdf]
