# Supplementary material for: TidyMass an object-oriented reproducible analysis framework for LC–MS data
Source: Nat Commun. 2022 Jul 28;13:4365. doi: 10.1038/s41467-022-32155-w (PMC9334349; doi:10.1038/s41467-022-32155-w)
Supplement: Supplementary file 6 — Reporting Summary [file 41467_2022_32155_MOESM6_ESM.pdf]

nature portfolio

Corresponding author(s): Michael P. Snyder, Caroline H. Johnson

Last updated by author(s): Jul 13, 2022

Reporting Summary

Nature Portfolio wishes to improve the reproducibility of the work that we publish. This form provides structure for consistency and transparency in reporting. For further information on Nature Portfolio policies, see our Editorial Policies and the Editorial Policy Checklist.

Statistics

For all statistical analyses, confirm that the following items are present in the figure legend, table legend, main text, or Methods section.

n/a	Confirmed	
☐	☒	The exact sample size (n) for each experimental group/condition, given as a discrete number and unit of measurement
☐	☒	A statement on whether measurements were taken from distinct samples or whether the same sample was measured repeatedly
☐	☒	The statistical test(s) used AND whether they are one- or two-sided<br>*Only common tests should be described solely by name; describe more complex techniques in the Methods section.*
☐	☒	A description of all covariates tested
☒	☐	A description of any assumptions or corrections, such as tests of normality and adjustment for multiple comparisons
☒	☐	A full description of the statistical parameters including central tendency (e.g. means) or other basic estimates (e.g. regression coefficient) AND variation (e.g. standard deviation) or associated estimates of uncertainty (e.g. confidence intervals)
☒	☐	For null hypothesis testing, the test statistic (e.g. F, t, r) with confidence intervals, effect sizes, degrees of freedom and P value noted<br>*Give P values as exact values whenever suitable.*
☒	☐	For Bayesian analysis, information on the choice of priors and Markov chain Monte Carlo settings
☒	☐	For hierarchical and complex designs, identification of the appropriate level for tests and full reporting of outcomes
☒	☐	Estimates of effect sizes (e.g. Cohen's d, Pearson's r), indicating how they were calculated

Our web collection on statistics for biologists contains articles on many of the points above.

Software and code

Policy information about availability of computer code

Data collection	ProteoWizard: Version. 3.0.19314-fb982f15b
Data analysis	R: 4.1.2; Rstudio: 2021.09.2. R package: colorspace_2.0-2 rjson_0.2.21 ellipsis_0.3.2 leaflet_2.1.0 rprojroot_2.0.2 circlize_0.4.14 GlobalOptions_0.1.2 clue_0.3-60 rstudioapi_0.13 mzR_2.28.0 affyio_1.64.0 fansi_1.0.2 xml2_1.3.3 codetools_0.2-18 ncdf4_1.19 doParallel_1.0.17 impute_1.68.0 knitr_1.37 jsonlite_1.7.3 cluster_2.1.2 vsn_3.62.0 png_0.1-7 readr_2.1.2 compiler_4.1.2 httr_1.4.2 assertthat_0.2.1 fastmap_1.1.0 lazyeval_0.2.2 limma_3.50.0 cli_3.2.0 htmltools_0.5.2 tools_4.1.2 gtable_0.3.0 glue_1.6.1 affy_1.72.0 dplyr_1.0.8 Biobase_2.54.0 cellranger_1.1.0 jquerylib_0.1.4 iterators_1.0.14 crosstalk_1.2.0 stringr_1.4.0 openxlsx_4.2.5 MSnbase_2.20.4 pcaMethods_1.86.0 hms_1.1.1 ProtGenerics_1.26.0 parallel_4.1.2 RColorBrewer_1.1-2 ComplexHeatmap_2.10.0 yaml_2.3.4 pbapply_1.5-0 yulab.utils_0.0.4 sass_0.4.0 stringi_1.7.6 highr_0.9 S4Vectors_0.32.3 foreach_1.5.2 BiocGenerics_0.40.0 zip_2.2.0 BiocParallel_1.28.3 shape_1.4.6 systemfonts_1.0.3 rlang_1.0.1 pkgconfig_2.0.3 matrixStats_0.61.0 mzID_1.32.0 evaluate_0.15 lattice_0.20-45 purrr_0.3.4 htmlwidgets_1.5.4 tidyselect_1.1.1 here_1.0.1 ggsci_2.9 plyr_1.8.6 bookdown_0.24 R6_2.5.1 IRanges_2.28.0 generics_0.1.2 DBI_1.1.2 pillar_1.7.0 withr_2.4.3 MsCoreUtils_1.6.0 tibble_3.1.6 crayon_1.5.0 utf8_1.2.2 plotly_4.10.0 tzdb_0.2.0 readxl_1.3.1 data.table_1.14.2 webshot_0.5.2 digest_0.6.29 tidyr_1.2.0 gridGraphics_0.5-1 ggplotify_0.1.0 bslib_0.3.1

For manuscripts utilizing custom algorithms or software that are central to the research but not yet described in published literature, software must be made available to editors and reviewers. We strongly encourage code deposition in a community repository (e.g. GitHub). See the Nature Portfolio guidelines for submitting code & software for further information.

Data

Policy information about availability of data

All manuscripts must include a data availability statement. This statement should provide the following information, where applicable:

- Accession codes, unique identifiers, or web links for publicly available datasets
- A description of any restrictions on data availability
- For clinical datasets or third party data, please ensure that the statement adheres to our policy

All the demo data for how to use tidyMass can be accessible on the tidyMass website (https://www.tidymass.org/), and it took about 8 hours to run the code on Mac OS with 32 GB RAM. For the case study, mass spectrometry raw converted data (mzML) for the case study in this paper is accessible on MetaboLights with MTBLS1122 (HILIC positive), MTBLS1124 (HILIC negative), MTBLS1122 (RPLC positive), and MTBLS1130 (RPLC negative). The MS2 data (mgf) and processed data (“mass_dataset” class) from the massProcesser package are available on the tidyMass project website (https://www.tidymass.org/start/case_study/), and the “mass_dataset” objects are provided as Supplementary Data 1. The in-house library used for metabolite annotation in the case study is provided based on your request.

Human research participants

Policy information about studies involving human research participants and Sex and Gender in Research.

Reporting on sex and gender	N/A
Population characteristics	N/A
Recruitment	N/A
Ethics oversight	N/A

Note that full information on the approval of the study protocol must also be provided in the manuscript.

Field-specific reporting

Please select the one below that is the best fit for your research. If you are not sure, read the appropriate sections before making your selection.

☒ Life sciences ☐ Behavioural & social sciences ☐ Ecological, evolutionary & environmental sciences

For a reference copy of the document with all sections, see nature.com/documents/nr-reporting-summary-flat.pdf

Life sciences study design

All studies must disclose on these points even when the disclosure is negative.

Sample size	In this study, we only re-analyzed data from a previously published study (Cai, Y. et al. Sex Differences in Colon Cancer Metabolism Reveal A Novel Subphenotype. Scientific Reports vol. 10 (2020)) and no new LC-MS data were produced.<br>39 normal colon tissues and 197 tumor tissue samples. No sample-size calculation was performed here. The sample sizes were chosen because they are the commonly used number for most biological studies in metabolomics society.
Data exclusions	Data were not excluded from analysis.
Replication	No replication in this study. The datasets is from public database about human cancer study, so it doesn't have replications like the animal study.
Randomization	For all datasets, samples were assigned randomly to acquire LC-MS data.
Blinding	The investigators were blinded to group allocation during data collection. At the time of sample acquisition and processing, scientists were completely unaware of the sample group. The data analyses were blinded.

Reporting for specific materials, systems and methods

We require information from authors about some types of materials, experimental systems and methods used in many studies. Here, indicate whether each material, system or method listed is relevant to your study. If you are not sure if a list item applies to your research, read the appropriate section before selecting a response.

Materials & experimental systems

n/a	Involved in the study
☒	☐ Antibodies
☒	☐ Eukaryotic cell lines
☒	☐ Palaeontology and archaeology
☒	☐ Animals and other organisms
☒	☐ Clinical data
☒	☐ Dual use research of concern

Methods

n/a	Involved in the study
☒	☐ ChIP-seq
☒	☐ Flow cytometry
☒	☐ MRI-based neuroimaging
